# Inhibition of HIF-prolyl hydroxylases improves healing of intestinal anastomoses

**DOI:** 10.1172/jci.insight.139191

**Published:** 2021-03-30

**Authors:** Moritz J. Strowitzki, Gwendolyn Kimmer, Julian Wehrmann, Alina S. Ritter, Praveen Radhakrishnan, Vanessa M. Opitz, Christopher Tuffs, Marvin Biller, Julia Kugler, Ulrich Keppler, Jonathan M. Harnoss, Johannes Klose, Thomas Schmidt, Alfonso Blanco, Cormac T. Taylor, Martin Schneider

**Affiliations:** 1 Department of General, Visceral and Transplantation Surgery, Heidelberg University, Heidelberg, Germany.; 2 School of Medicine and Conway Institute of Biomolecular & Biomedical Research, University College Dublin, Dublin, Ireland.; 3 Department of Anaesthesiology, Heidelberg University, Heidelberg, Germany.; 4 Flow Cytometry Core Technology. Conway Institute of Biomolecular & Biomedical Research, University College Dublin, Dublin, Ireland.

**Keywords:** Gastroenterology, Inflammation, Collagens, Hypoxia, Macrophages

## Abstract

Anastomotic leakage (AL) accounts for a major part of in-house mortality in patients undergoing colorectal surgery. Local ischemia and abdominal sepsis are common risk factors contributing to AL and are characterized by upregulation of the hypoxia-inducible factor (HIF) pathway. The HIF pathway is critically regulated by HIF-prolyl hydroxylases (PHDs). Here, we investigated the significance of PHDs and the effects of pharmacologic PHD inhibition (PHI) during anastomotic healing. Ischemic or septic colonic anastomoses were created in mice by ligation of mesenteric vessels or lipopolysaccharide-induced abdominal sepsis, respectively. Genetic PHD deficiency (*Phd1*^–/–^, *Phd2^+/–^*, and *Phd3*^–/–^) or PHI were applied to manipulate PHD activity. Pharmacologic PHI and genetic PHD2 haplodeficiency (*Phd2^+/–^*) significantly improved healing of ischemic or septic colonic anastomoses, as indicated by increased bursting pressure and reduced AL rates. Only *Phd2^+/–^* (but not PHI or *Phd1*^–/–^) protected from sepsis-related mortality. Mechanistically, PHI and *Phd2^+/–^* induced immunomodulatory (M2) polarization of macrophages, resulting in increased collagen content and attenuated inflammation-driven immune cell recruitment. We conclude that PHI improves healing of colonic anastomoses in ischemic or septic conditions by *Phd2^+/–^*-mediated M2 polarization of macrophages, conferring a favorable microenvironment for anastomotic healing. Patients with critically perfused colorectal anastomosis or abdominal sepsis could benefit from pharmacologic PHI.

## Graphical abstract

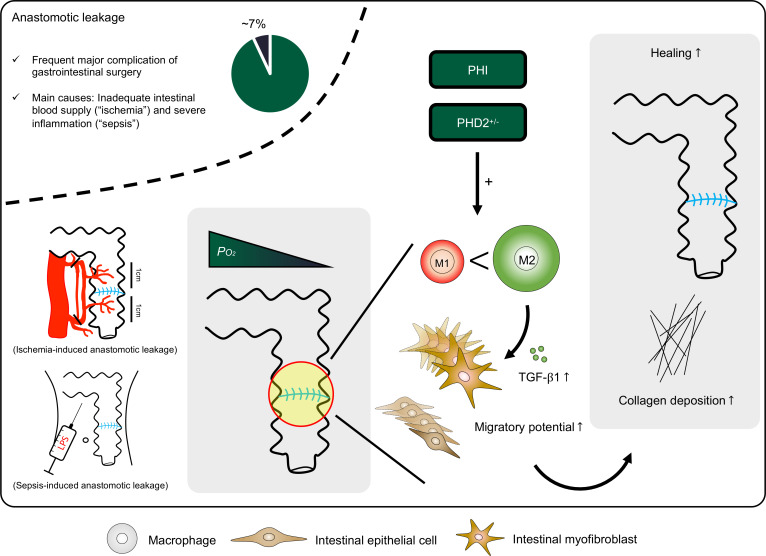

## Introduction

Anastomotic leakage (AL) occurs in up to 7% of all patients undergoing colorectal surgery (1). Despite the obvious clinical and economic burden (2), currently no reliable treatment options exist to improve healing of intestinal anastomoses and, thus, to prevent AL (3).

Sufficient oxygen supply to intestinal anastomoses is paramount for healing (4, 5), and ischemia represents one main cause for AL after colorectal surgery (6). Perioperative abdominal sepsis, which leads to profound tissue inflammation, can further aggravate intestinal hypoxia (an imbalance between high oxygen demand and limited oxygen supply) (7). Indeed, infiltrating immune cells consume high amounts of oxygen and can thus promote AL (8–10).

In hypoxic conditions, all metazoans that rely on sufficient oxygen supply are able to initiate a specific adaptive gene program, called the hypoxia-inducible factor (HIF) pathway (11). This molecular response involves upregulation of numerous HIF target genes, which in concert either increase oxygen supply, for example, by forming new blood vessels (angiogenesis) and red blood cells (erythropoiesis), or adapt cellular metabolism to decrease oxygen consumption (11). In normoxia, HIF-prolyl hydroxylases (PHD1, PHD2, and PHD3) utilize oxygen to enzymatically target HIFα subunits for proteasomal degradation, thereby preventing the expression of HIF target genes. Importantly, the HIF pathway can be induced by pharmacologic PHD inhibitors (PHIs), such as dimethyloxalylglycine (DMOG), which inhibit the enzymatic function of PHDs (12, 13). The first PHI has recently been approved to treat anemia in patients with chronic kidney diseases (14).

Both wound healing and inflammation are hallmarked by hypoxia (9), and the HIF pathway is involved in macrophage activation and polarization (M1 versus M2), which occurs when macrophages leave the oxygen-rich bloodstream to migrate into inflamed, oxygen-deprived organs or tissues (13, 15). In this context, it has recently been shown that loss of *Phd1* induces immunomodulatory (M2) macrophages, which in contrast to proinflammatory (M1) macrophages elicit a favorable (pro–wound healing) microenvironment (9), thus attenuating experimental colitis in mice (16). Notably, *Phd2* haplodeficiency has likewise been shown to stimulate polarization of M2 macrophages, thereby reducing necrosis in a murine hind limb ischemia model (17). In addition, there is compelling evidence that loss of *Phd1* and PHIs reduce inflammation and disease activity in murine colitis (9, 18, 19).

Taken together, PHDs are a promising therapeutic target to treat hypoxia- and inflammation-driven processes, including intestinal (wound) healing. We, therefore, aimed to determine the role of PHDs and PHIs during healing of intestinal anastomoses and macrophage polarization under challenging conditions, such as ischemia and abdominal sepsis.

## Results

### Pharmacologic HIF-prolyl hydroxylase inhibition improves healing of ischemic colonic anastomoses.

**Figure 1:**
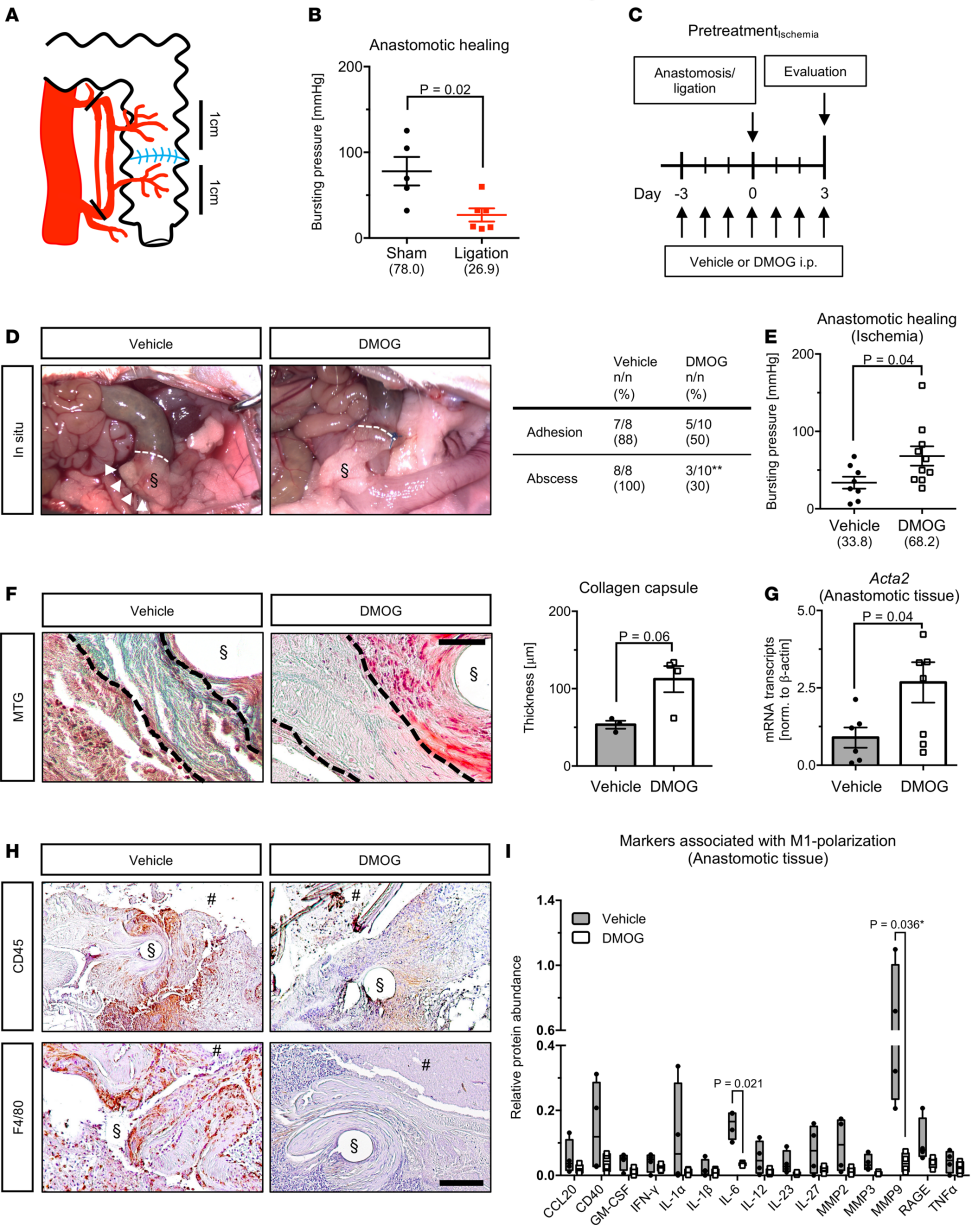
Pharmacologic inhibition of HIF-prolyl hydroxylases improves healing of ischemic anastomoses. (**A**) Schematic drawing of blood vessel ligation to induce ischemia in colonic anastomoses. (**B**) Bursting pressure analysis of ischemic (ligation, red dots) and nonischemic colonic anastomoses (sham, black dots). (**C**) Schematic overview of pretreatment with PHI (DMOG, 100 μg/g BW) or vehicle control in vivo. (**D**) *Left:* Representative images of ischemic colonic anastomoses (dotted white lines) from vehicle- and DMOG-treated mice. Note indirect signs of anastomotic leakage, such as severe adhesion (§) and abscess (white arrowheads). *Right:* Quantification of indirect signs of anastomotic leakage (*n* = 8–10 animals per group; **P = 0.003 by χ^2^ test). (**E**) Bursting pressure analysis of ischemic colonic anastomoses from vehicle- and DMOG-treated mice (*n* = 8–10 animals per group; Student’s *t* test). (**F**) *Left:* Representative Masson-Trichrome-Goldner (MTG) stainings, revealing the thickness of collagen capsules (black dashed lines) close to sutures (§) in ischemic anastomoses from vehicle- and DMOG-treated mice (scale bar represents 100 μm). *Right:* Histomorphometric quantification of collagen capsule thickness in ischemic colonic anastomoses from vehicle- and DMOG-treated mice (*n* = 3–4 animals per group; Mann-Whitney *U* test). (**G**) Real-time PCR analysis of *Acta2* mRNA expression within ischemic anastomoses from vehicle- and DMOG-treated mice (*n* = 3–4 biological replicates; Student’s *t* test). (**H**) Representative CD45 (upper panels) and F4/80 (lower panels) immunostainings of ischemic colonic anastomoses from vehicle- and DMOG-treated mice (*n* = 3–4 animals per group; scale bar represents 200 μm; § and # in **F** and **H** indicate positions of [extracted] sutures and gut lumen, respectively). (**I**) Antibody array, revealing the relative protein abundance of M1 macrophage–associated cytokines within whole tissue from vehicle- and DMOG-treated ischemic colonic anastomoses (*n* = 4 biological replicates; *nonadjusted *P* value reported, after correction for multiple comparison with Holm-Šidák method, *P* = 0.402).

Since local ischemia is a main driver of AL (4), we established a mouse model of ischemia-inflicted AL to determine the impact of DMOG-mediated PHD inhibition on healing of ischemic colonic anastomoses (20). Ligation of critical blood vessels 1 cm proximal and distal of colonic anastomoses (Figure 1A) resulted in a significant reduction of anastomotic bursting pressure compared with sham operation, confirming ischemia-impaired healing (Figure 1B). Blood sample analysis on day 3 suggested increased erythropoiesis in PHI-treated animals compared with vehicle-treated controls (Supplemental Table 1; supplemental material available online with this article; https://doi.org/10.1172/jci.insight.139191DS1), indicating systemic stimulation of HIF signaling. Quantification of HIF target gene mRNA levels and HIF-1α protein levels within colonic anastomotic tissue additionally confirmed local upregulation of the HIF pathway by the PHI (Supplemental Figure 1, A and B). Notably, PHI pretreatment markedly reduced indirect signs of AL (such as adhesion and abscess formation) and increased bursting pressure, indicating improved healing of ischemic colonic anastomoses (Figure 1, C–E). Under nonischemic conditions, however, PHD inhibition did not affect anastomotic healing (Supplemental Figure 4A).

Collagen deposition driven by intestinal myofibroblasts is a major factor determining sufficient tissue strength and, thus, anastomotic healing (21). We therefore performed Masson-Trichrome-Goldner (MTG) staining to detect collagen deposition in proximity to anastomotic suture lines (Supplemental Figure 2A). Indeed, the thickness of the collagen capsule correlated with bursting pressure of colonic anastomoses in all mice (Supplemental Figure 5A). Immunostaining of activated α–smooth muscle actin–positive (αSMA-positive) myofibroblasts, which regulate collagen deposition (22), within colonic anastomoses likewise revealed a strong correlation between myofibroblast numbers and bursting pressure (Supplemental Figure 5A). Strikingly, collagen capsule thickness was increased, and mRNA transcript levels of *Acta2* (encoding αSMA protein) were enhanced in ischemic colonic anastomoses from PHI-treated mice compared with vehicle-treated controls (Figure 1, F and G).

Local hypoxia due to reduced numbers of blood vessels per se may affect collagen synthesis and degradation (9). Surprisingly, PHD inhibition did not significantly affect the number of blood vessels (CD31-positive cells) or the severity of hypoxia (pimonidazole-positive cells) within ischemic colonic anastomoses (Supplemental Figure 2A and Supplemental Figure 6A).

In search of alternative mechanisms underlying the PHI-elicited increase in collagen capsule thickness, we determined the number of proinflammatory cells, which degrade collagen within colonic anastomoses (21). Indeed, in all animals, immunostaining and histomorphometric quantification of CD45- or F4/80-positive cells revealed a negative correlation between collagen capsule thickness and leukocyte or macrophage infiltration, respectively (Supplemental Figure 5B). Importantly, PHI pretreatment significantly reduced the numbers of CD45- and F4/80-positive cells within ischemic colonic anastomoses (Figure 1H and Supplemental Figure 7A). This PHI-mediated decrease in leukocyte and macrophage infiltration was accompanied by a marked decrease in protein expression of various proinflammatory cytokines, such as interleukin 6 (IL-6), as detected by antibody array in tissue lysates from ischemic anastomotic tissue (Figure 1I). Interestingly, protein expression of several collagen-degrading enzymes that are known to impair healing of intestinal anastomoses, including matrix metalloproteinase 9 (20, 23), and chemokines that could attract other proinflammatory cells were likewise reduced upon PHI pretreatment (Figure 1I and Supplemental Figure 7B).

Taken together, PHI improved healing of ischemic colonic anastomoses, probably by decreasing the ischemia-inflicted proinflammatory response. PHI augmented anastomotic collagen content, thereby strengthening tissue integrity within ischemic colonic anastomoses.

### Significance of individual HIF-prolyl hydroxylases in anastomotic healing.

To determine the significance of individual PHD enzymes in anastomotic healing, colonic anastomoses were created in WT, Sw/129-TgN(Egln2)Uhg (*Phd1^–/–^*), Sw/129-TgN(Egln1)Uhg (*Phd2*^+/–^), and Sw/129-TgN(Egln3)Uhg (*Phd3^–/–^*) mice. Consistent with results upon PHI pretreatment, anastomotic bursting pressure was comparable in *Phd1^–/–^*, *Phd2*^+/–^, and *Phd3^–/–^* animals at baseline conditions (Supplemental Figure 4B). However, indirect signs of AL (such as adhesion and abscess formation), and gross structural anastomotic defects (“anastomotic insufficiencies”) were markedly increased in *Phd3*-deficient mice at baseline conditions (Supplemental Figure 4C). In subsequent experiments, we therefore focused on *Phd1^–/–^* and *Phd2^+/–^*.

**Figure 2:**
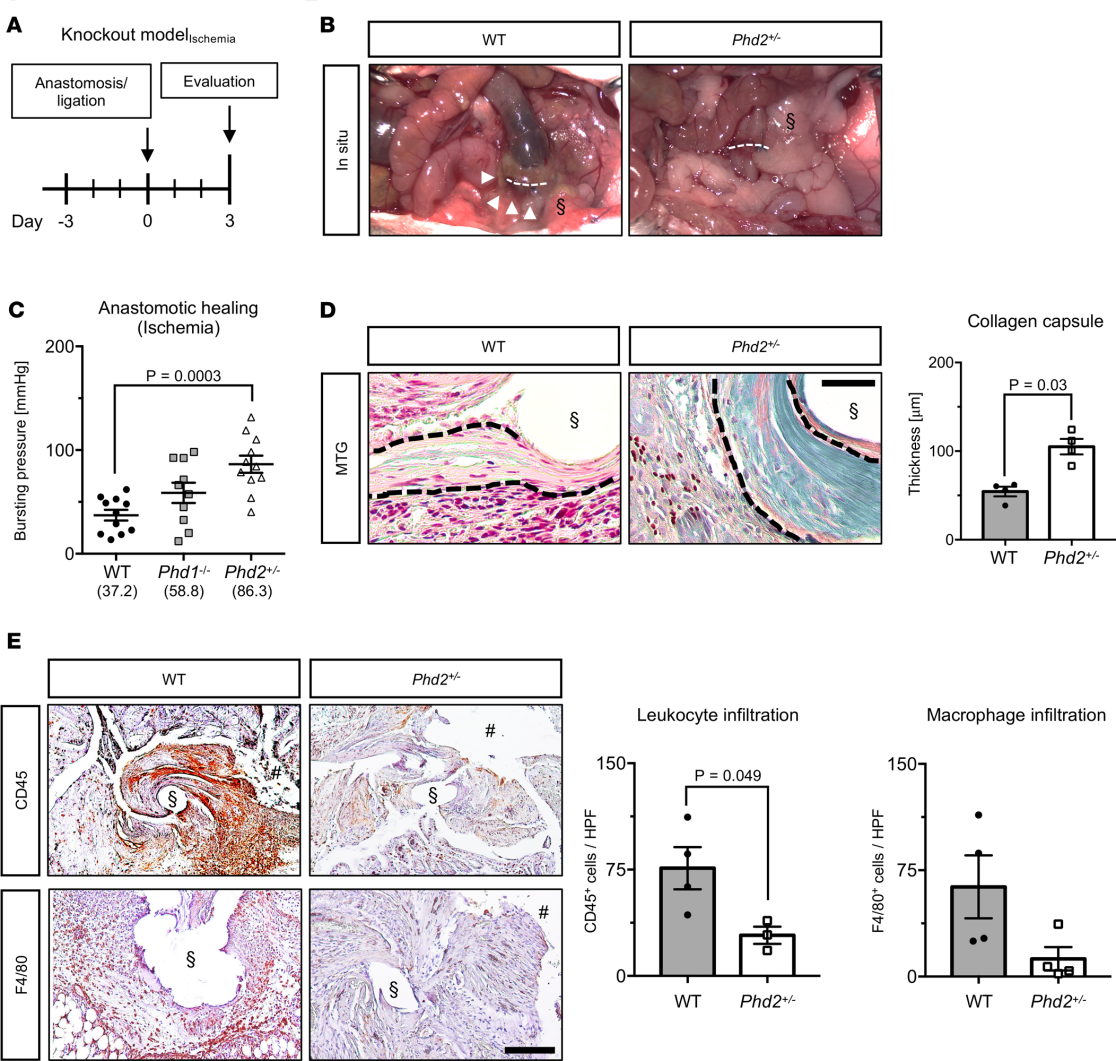
Haplodeficiency of HIF-prolyl hydroxylase 2 augments healing of ischemic colonic anastomoses. (**A**) Experimental schedule. Surgical anastomosis and blood vessel ligation were performed in WT, *Phd1*-deficient (*Phd1^–/–^*), and *Phd2*-haplodeficient (*Phd2^+/–^*) mice, and evaluation was performed 3 days thereafter. (**B**) Representative images of ischemic colonic anastomoses (dotted white lines) from WT and *Phd2^+/–^* mice. Note indirect signs of AL, such as severe adhesion (§) and abscess (white arrowheads). (**C**) Bursting pressure analysis of ischemic colonic anastomoses harvested from WT, *Phd1^–/–^*, and *Phd2^+/–^* mice (*n* = 10–11 animals per group; ANOVA with post hoc test). (**D**) *Left:* Representative MTG stainings, revealing the thickness of collagen capsules (black dashed lines) close to sutures (§) in ischemic anastomoses harvested from WT and *Phd2^+/–^* mice (scale bar represents 100 μm). *Right:* Histomorphometric quantification of collagen capsule thickness in ischemic colonic anastomoses from WT and *Phd2^+/–^* mice (*n* = 4 animals per group; Mann-Whitney *U* test). (**E**) Representative immunolabeling of leukocytes (CD45, *left* *upper panel*) and macrophages (F4/80, *left lower panel*) in ischemic anastomoses from WT and *Phd2^+/–^* mice and histomorphometric quantification (*right*) (*n* = 3–4 animals per group; scale bar represents 200 μm; § and # indicate positions of [extracted] sutures and gut lumen, respectively). HPF, high-power field.

We next evaluated the significance of PHD1 and PHD2 during healing of ischemic colonic anastomoses (Figure 2A). Notably, indirect signs of AL were significantly reduced (*P* < 0.05) in both *Phd1^–/–^* and *Phd2^+/–^* mice after ischemia (Figure 2B and Supplemental Figure 4D). However, while *Phd1* deficiency only slightly increased bursting pressure values upon ischemia, *Phd2* haplodeficiency significantly improved the bursting pressure of ischemic anastomoses (Figure 2C). Moreover, MTG staining revealed that the thickness of collagen capsules within ischemic colonic anastomoses was markedly increased in *Phd2^+/–^* mice compared with WT animals (Figure 2D). Increased collagen deposition in ischemic anastomoses from *Phd2^+/–^* mice occurred concomitantly with reduced infiltration by CD45-positive leukocytes and F4/80-positive macrophages (Figure 2E), whereas the density of CD31-positive blood vessels and severity of hypoxia (as assessed by pimonidazole staining) were comparable in ischemic anastomoses from WT and *Phd2*^+/–^ animals (Supplemental Figure 6B).

Taken together, PHD2 haplodeficiency significantly enhanced anastomotic healing in ischemic conditions. Beneficial effects of PHD1 deficiency (*Phd1^–/–^*) appeared more subtle, while PHD3 deficiency (*Phd3^–/–^*) might even provoke opposite effects.

### Effects of pharmacologic PHD inhibition and PHD deficiency on anastomotic healing in septic conditions.

**Figure 3:**
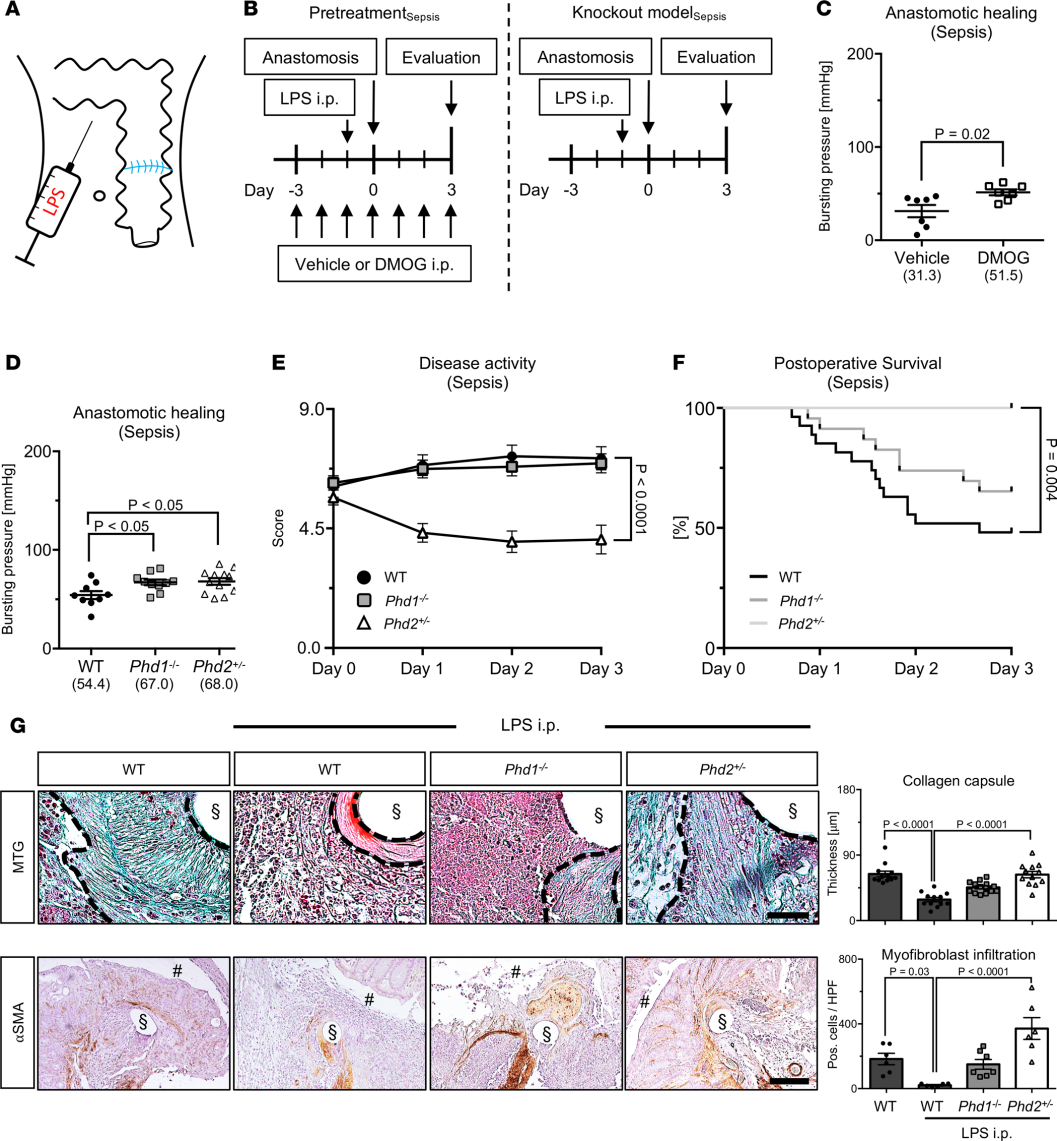
Pharmacologic and genetic inhibition of HIF-prolyl hydroxylases improves healing of septic colonic anastomoses. (**A** and **B**) Schematic drawing (**A**) and experimental schedule (**B**). LPS was injected i.p. 18 hours prior to creation of anastomoses. Pretreatment with PHI (DMOG, 100 μg/g BW) or vehicle control was performed for 3 days prior to anastomosis and continued for 7 days until evaluation (**B**, left). Alternatively, LPS injection and surgical anastomosis was performed in WT, *Phd1^–/–^*, and *Phd2^+/–^* mice, and evaluation was performed 3 days thereafter (**B**, right). (**C** and **D**) Bursting pressure analysis of septic colonic anastomoses from vehicle- or DMOG-treated mice (**C**) or from WT, *Phd1^–/–^*, and *Phd2^+/–^* mice (**D**) (pretreatment: *n* = 7 animals per group; Student’s *t* test; knockout model: *n* = 9–12 animals per group; ANOVA with post hoc test). (**E** and **F**) Postoperative disease activity (**E**) and survival (**F**) of WT, *Phd1^–/–^*, and *Phd2^+/–^* mice on days 0–3 after LPS-induced sepsis and colonic anastomosis. Note significantly attenuated disease activity and improved survival in *Phd2^+/–^* mice (*n* = 12–27 animals per group; differences in disease activity score in **E** were analyzed by 2-way ANOVA with post hoc test and survival curves in **F** by log-rank test). (**G**) Representative MTG stainings of collagen capsules (black dashed lines in *left upper panels*) and immunolabeling of myofibroblasts (αSMA, *left* *lower panels*) in healthy and septic (LPS i.p.) anastomoses from WT, *Phd1^–/–^*, and *Phd2^+/–^* mice and histomorphometric quantification (*right*) (*n* = 6–7 animals per group; ANOVA or Kruskal-Wallis test where appropriate; scale bars in top and bottom panels represent 100 μm and 200 μm, respectively; § and # indicate positions of [extracted] sutures and gut lumen, respectively).

Next to local ischemia, severe inflammation due to perioperative abdominal sepsis is a major cause of AL after colorectal surgery (10, 24). To assess the significance of PHD inhibition and PHD deficiency in this context, we established a mouse model of anastomotic healing upon LPS-inflicted abdominal sepsis (Figure 3, A and B). Analysis of anastomotic bursting pressure following i.p. injection of LPS at increasing dosages revealed that the lowest dosage of 0.1 mg/kg BW did not affect healing of colonic anastomoses or postoperative survival (Supplemental Figure 8, A and B), whereas LPS injection at a dosage of 0.5 mg/kg BW significantly reduced anastomotic bursting pressure and markedly impaired postoperative survival (Supplemental Figure 8, A and B). In subsequent experiments, we therefore applied i.p. injections of 0.5 mg/kg BW LPS to mimic anastomotic healing in septic conditions.

Pharmacologic PHI pretreatment significantly improved bursting pressure of colonic anastomoses in septic conditions, however, without affecting postoperative survival (Figure 3B, left; Figure 3C; and Supplemental Figure 8C). Next, we evaluated the significance of PHD1 and PHD2 in healing of septic colonic anastomoses (Figure 3B, right). Whereas in ischemic conditions significantly enhanced anastomotic healing was observed only in *Phd2^+/–^* but not *Phd1^–/–^* mice (see above and Figure 2C), bursting pressure of septic anastomoses was significantly enhanced in both *Phd1^–/–^* and *Phd2^+/–^* animals (Figure 3D). However, daily measurement of a compound disease activity index (DAI, ref. 25) revealed that the severity of clinical sepsis symptoms provoked by combined LPS treatment and colonic anastomosis was markedly attenuated in *Phd2^+/–^* mice compared with their WT and *Phd1^–/–^* counterparts (Figure 3E). Consistently, survival upon anastomosis construction in septic conditions was significantly impaired in WT and *Phd1^–/–^* but not in *Phd2^+/–^* mice (Figure 3F). Collectively, these data reveal significantly improved healing of septic colonic anastomoses in *Phd2^+/–^* mice.

MTG staining and histomorphometric quantification of collagen capsules revealed that LPS treatment reduced collagen deposition within colonic anastomoses of WT mice compared with treatment with vehicle control (Figure 3G, top panels). Notably, *Phd2* haplodeficiency, but not *Phd1* deficiency, abrogated this LPS-inflicted decrease in collagen deposition within septic colonic anastomoses (Figure 3G, top panels). Consistently, numbers of αSMA-positive myofibroblasts were increased within septic colonic anastomoses of *Phd2^+/–^* mice compared with those of WT and *Phd1^–/–^* animals (Figure 3G, bottom panels).

In summary, PHD inhibition and *Phd2^+/–^* significantly improved healing of septic colonic anastomoses. A trend toward a similar effect was observed in *Phd1^–/–^* mice but was less pronounced than in *Phd2^+/–^* animals.

### PHD2 modulates innate immune responses during healing of septic anastomoses.

**Figure 4:**
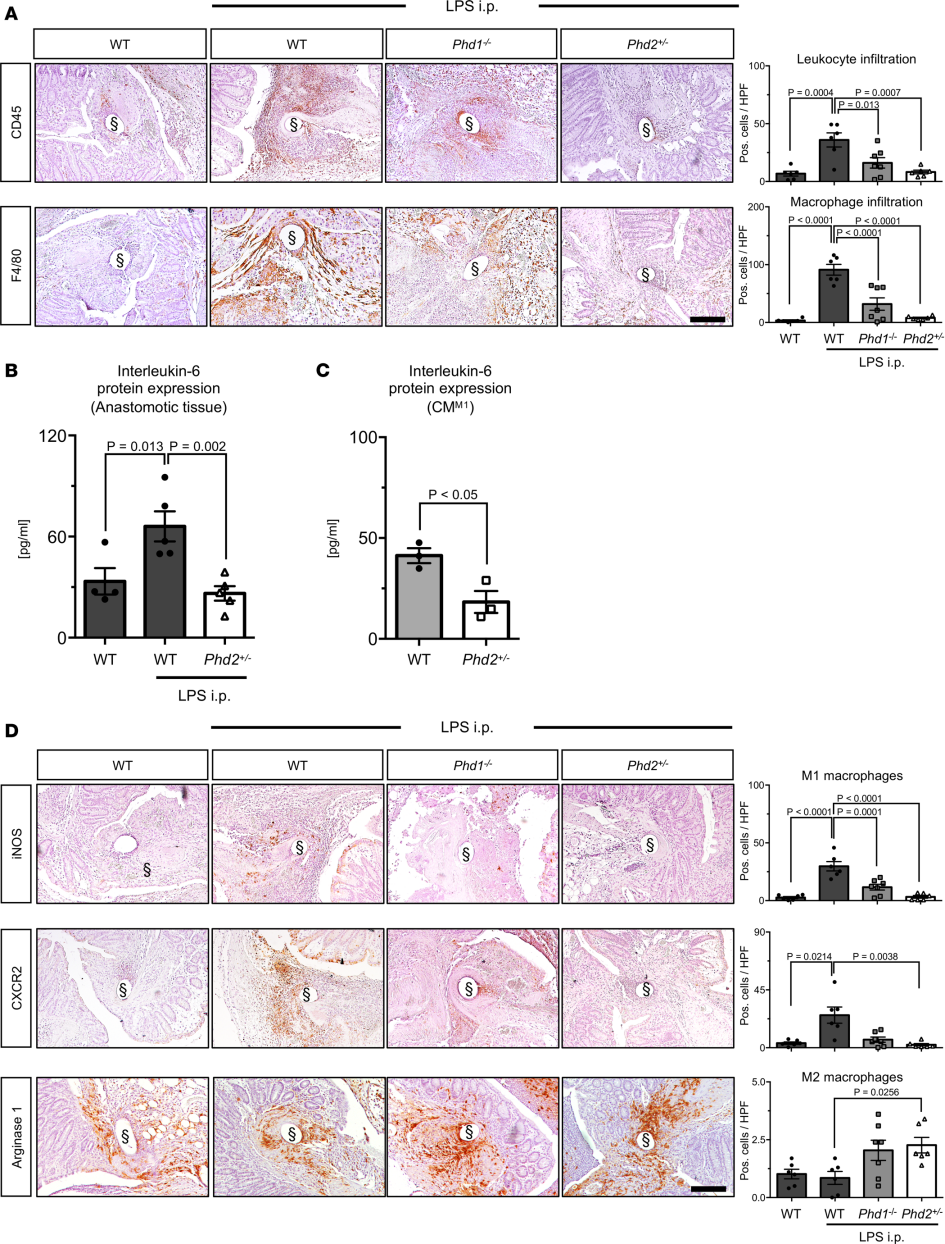
Phd2 haplodeficiency reduces recruitment of inflammatory cells and enhances M2 macrophages during healing of septic colonic anastomoses. (**A**) Representative immunolabeling of leukocytes (CD45, *left* *upper panels*) and macrophages (F4/80, *left lower panels*) in healthy and septic (LPS i.p.) anastomoses from WT, *Phd1^–/–^*, and *Phd2^+/–^* mice and histomorphometric quantification (*right*). (**B** and **C**) ELISA-based quantification of IL-6 in whole tissue lysates from healthy and septic (LPS i.p.) anastomoses harvested from WT and *Phd2^+/–^* mice (**B**) or in cell culture medium (CM^M1^) conditioned with LPS-treated M1-polarized primary WT or *Phd2^+/–^* BMDMs (**C**) (*n* = 3, pooled data from individual biological replicates; ANOVA with post hoc test in **B** and Student’s *t* test in **C**). (**D**) Representative immunolabeling of proinflammatory M1 macrophages (iNOS and CXCR2, *left* *upper panels*) and immunomodulatory M2 macrophages (Arginase 1, *left lower panels*) in healthy and septic (LPS i.p.) anastomoses from WT, *Phd1^–/–^*, and *Phd2^+/–^* mice and histomorphometric quantification (*right*) (in **A** and **D**: *n* = 6–7 animals per group; ANOVA with post hoc or Kruskal-Wallis [only CXRC2] test; scale bar represents 200 μm; § indicates positions of [extracted] sutures).

Immunostaining for CD45 and F4/80 revealed that infiltration of leukocytes and macrophages, respectively, into anastomoses was starkly induced by pretreatment with LPS (Figure 4A, top and bottom panels). This LPS-elicited infiltration of innate immune cells into septic anastomoses was potently attenuated in *Phd2^+/–^* mice (Figure 4A, top and bottom panels), suggesting that PHD2 crucially modulates LPS-induced inflammatory responses in the context of anastomotic healing. Further cell culture experiments were carried out to substantiate this notion. Indeed, mRNA levels of the chemotactic cytokine CXC motif chemokine ligand 1 (*Cxcl1*) were significantly lower in LPS-treated bone marrow–derived macrophages (BMDMs) isolated from *Phd2^+/–^* mice than in BMDMs isolated from WT or *Phd1^–/–^* animals (Supplemental Figure 9A). Consistently, ELISA-based detection revealed lower CXCL-1 protein levels within conditioned medium from LPS-treated *Phd2^+/–^* M1-polarized BMDMs (CM^M1^) compared with medium conditioned with WT or *Phd1^–/–^* BMDMs, albeit this difference did not reach statistical significance (Supplemental Figure 9B). In accordance with the role of IL-6 in inflammatory cell recruitment and regulation of CXCL-1 expression (26, 27), LPS-induced expression of IL-6 protein was significantly attenuated both in tissue lysates of septic colonic anastomoses from *Phd2^+/–^* mice and in CM^M1^ harvested from *Phd2^+/–^* BMDMs (Figure 4, B and C). Notably, these results are consistent with our abovementioned observation that pharmacologic PHI pretreatment attenuates the expression of IL-6 and CXCL-1 within ischemic anastomoses (Figure 1I and Supplemental Figure 7B). Taken together, these findings underscore antiinflammatory properties of *Phd2* haplodeficiency in the context of anastomotic healing, which are likely conferred by reduced chemokine- and cytokine-elicited recruitment of inflammatory cells.

Another component of the innate immune system that modulates anastomotic healing is macrophage polarization. While proinflammatory (M1) macrophages have been associated with impaired healing of intestinal anastomoses, immunomodulatory (M2) macrophages improve healing (9, 28). We therefore analyzed whether PHD deficiency affects the composition of macrophage subsets (M1 versus M2). Immunostaining for the M1 macrophage markers inducible NO synthase (iNOS) and CXC chemokine receptor type 2 (CXCR2) and morphometric quantification revealed that the abundance of iNOS- and CXCR2-positive (M1) macrophages within septic anastomoses was significantly increased upon LPS exposure (Figure 4D, top and middle). Numbers of M1 macrophages were, however, significantly lower in septic anastomoses from *Phd1^–/–^* or *Phd2^+/–^* mice than in those from WT animals (Figure 4D, top and middle). Indeed, numbers of M1 macrophages in LPS-challenged *Phd2^+/–^* mice were almost comparable to numbers observed in WT animals without LPS stimulus (WT: 2.7, WT + LPS: 29.7, *Phd1^–/–^* + LPS: 11.6, *Phd2^+/–^* + LPS: 3.3; given in iNOS-positive cells/HPF). Conversely, although LPS exposure did not alter the abundance of arginase 1–positive (M2) macrophages within septic colonic anastomoses in WT mice, numbers of M2 macrophages were significantly increased in LPS-challenged anastomoses from *Phd2^+/–^* mice, suggesting that *Phd2* haplodeficiency induces a “switch” in macrophage polarization (fewer M1 than M2 macrophages) upon anastomotic healing in septic conditions (Figure 4D, bottom).

Collectively, these findings suggest that favorable effects of PHD2 haplodeficiency in healing of septic anastomoses are attributable to its effects on innate immune cells, specifically, to reduced inflammatory cell recruitment and macrophage skewing toward an M2 phenotype.

### Pharmacologic or genetic inhibition of PHD2 induces macrophage M2 polarization in vitro.

On the basis of the findings described above, we speculated that genetic PHD2 haplodeficiency and pharmacologic PHD inhibition augment anastomotic healing in ischemic and septic conditions by inducing immunomodulatory (M2) macrophages, thus conferring a favorable (pro–wound healing) microenvironment. To further substantiate this hypothesis, we analyzed how pharmacologic PHD inhibition or haplodeficiency of PHD2 (*Phd2^+/–^*) affects macrophage phenotypes upon treatment with LPS or IL-4 to induce M1 or M2 polarization, respectively. These analyses were carried out under both normoxia (21% O_2_) and hypoxia (0.75% O_2_) to better reflect the hypoxic microenvironment of anastomotic healing in ischemic or septic conditions.

**Figure 5:**
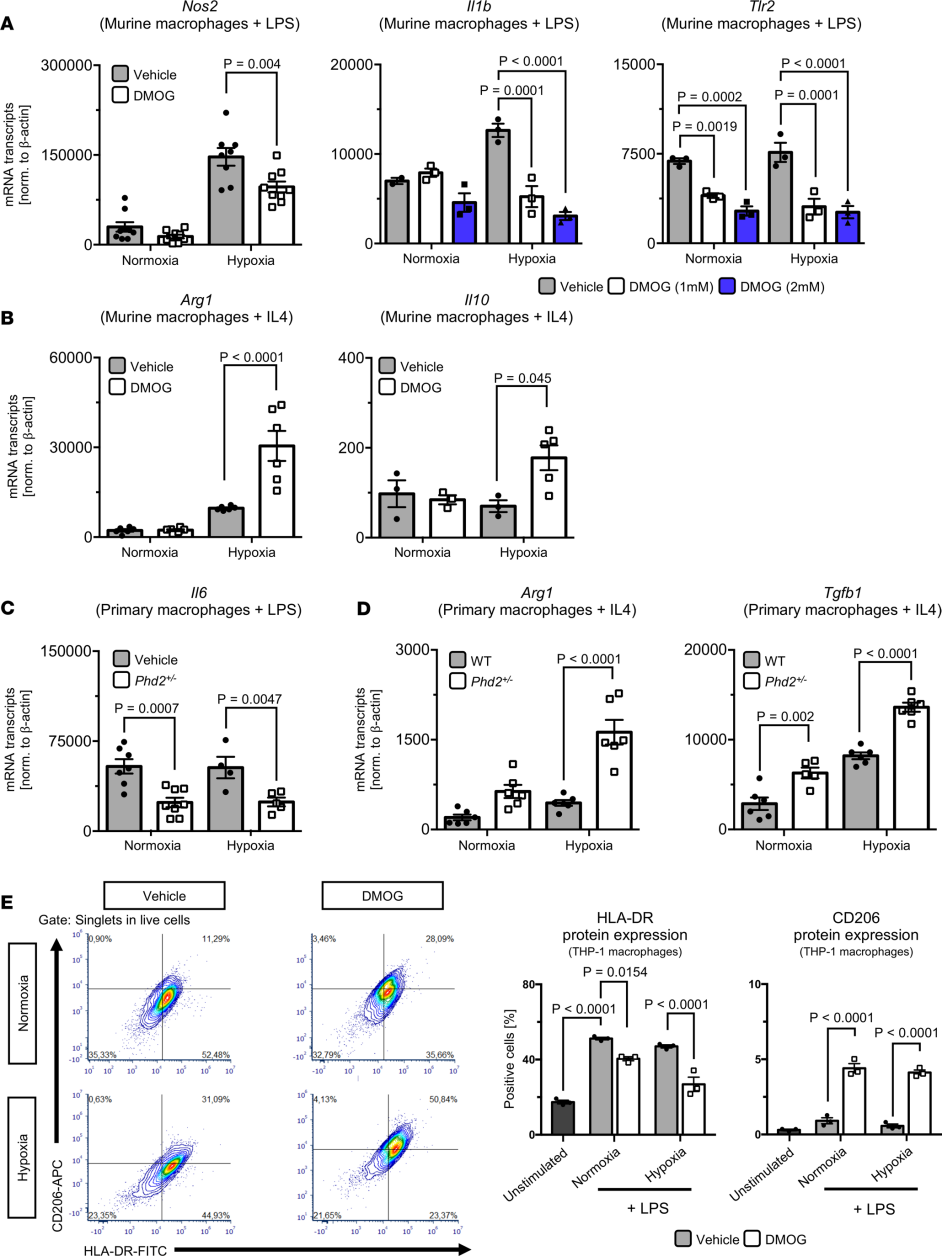
Pharmacologic and genetic inhibition of HIF-prolyl hydroxylases induces M2 macrophage polarization. (**A**–**D**) Real-time PCR analysis of proinflammatory M1 (**A** and **C**) and immunomodulatory M2 (**B** and **D**) markers in vehicle- or DMOG-treated (1 mM, if not otherwise indicated) murine macrophages (J774A.1 cell line; **A** and **B**) and primary WT or *Phd2^+/–^* bone marrow–derived macrophages (BMDMs; **C** and **D**) treated 24 hours with normoxia (21% oxygen) or hypoxia (0.75% oxygen) +/– LPS (100 ng/mL) or IL-4 (10 ng/mL) to induce macrophage polarization. (**E**) Flow cytometry to quantify protein expression of proinflammatory (M1) marker HLA-DR and M2 marker CD206 in THP-1 macrophages treated with normoxia (21% oxygen) or hypoxia (0.75% oxygen) +/– LPS (100 ng/mL) for 24 hours. Note that DMOG and *Phd2^+/–^* reduce proinflammatory (M1) markers and induce immunomodulatory (M2) markers, indicating PHD2-related M2 polarization, and that this effect is more pronounced in hypoxic conditions. ANOVA with post hoc test. All experiments were performed in biological triplicates.

Indeed, in hypoxia, PHD inhibition by DMOG significantly reduced mRNA expression of the M1 marker *Nos2* in LPS-stimulated murine macrophages (J774A.1 cell line), while this effect was less pronounced in normoxic conditions (Figure 5A, left). To address whether simultaneous hypoxia augmented the PHI-elicited reduction in M1 polarization, LPS-stimulated murine macrophages were treated with vehicle or increasing DMOG concentrations (1 mM and 2 mM) under normoxic and hypoxic conditions. Importantly, in normoxia, 2 mM DMOG significantly reduced the expression of the M1 marker Toll-like receptor 2 (*Tlr2*), while *Il1b* mRNA levels were not significantly changed (Figure 5A, right). In hypoxia, the DMOG-mediated relative reduction of both *Il1b* and *Tlr2* mRNA levels was further enhanced (Figure 5A, right). Conversely, the PHI increased mRNA expression of the M2 markers arginase 1 (*Arg1*) and *Il10* in J774A.1 macrophages stimulated with IL-4 under hypoxic conditions, while this effect was absent in normoxia (Figure 5B). In *Phd2^+/–^* BMDMs stimulated with LPS, *Il6* mRNA levels were downregulated in normoxia and hypoxia (Figure 5C). Expression levels of the M2 markers *Arg1* and TGF-β1 (*Tgfb1*; both involved in wound healing and collagen deposition) were significantly higher in *Phd2^+/–^* BMDMs than in corresponding WT BMDMs upon IL-4 stimulation (Figure 5D). *Phd2^+/–^*-related effects on the expression of M2 markers were only subtle in normoxia but pronounced in hypoxia (Figure 5D). Flow cytometry–based analysis of surface protein expression of the M1 marker HLA-DR and the M2 marker CD206 confirmed DMOG-mediated M2 polarization of LPS-stimulated THP-1 macrophages (Figure 5E). Comparable to the findings based on gene expression analysis in murine macrophages, the DMOG-inflicted decrease in M1 polarization was more pronounced in LPS-stimulated THP-1 macrophages treated with hypoxia (Figure 5E). Upregulation of the HIF pathway in DMOG-treated murine and human macrophages was confirmed by real-time PCR–based (RT-PCR–based) quantification of HIF target genes and quantification of HIF-1α protein levels by Western blotting, respectively (Supplemental Figure 9, C and D).

Since proinflammatory M1 macrophages are crucially involved in clearing bacteria from the inflamed site, we next evaluated whether PHD inhibition affects the phagocytotic capacity of macrophages. In vitro phagocytosis assays revealed that PHD inhibition by DMOG did not significantly alter phagocytosis of WT BMDMs under normoxia or hypoxia (Supplemental Figure 9E).

Collectively, these in vitro findings support the notion that pharmacologic PHD inhibition and genetic PHD2 haplodeficiency induce M2 polarization of macrophages, particularly in hypoxic conditions.

### Pharmacologic or genetic inhibition of PHD2 induces wound healing in vitro.

**Figure 6:**
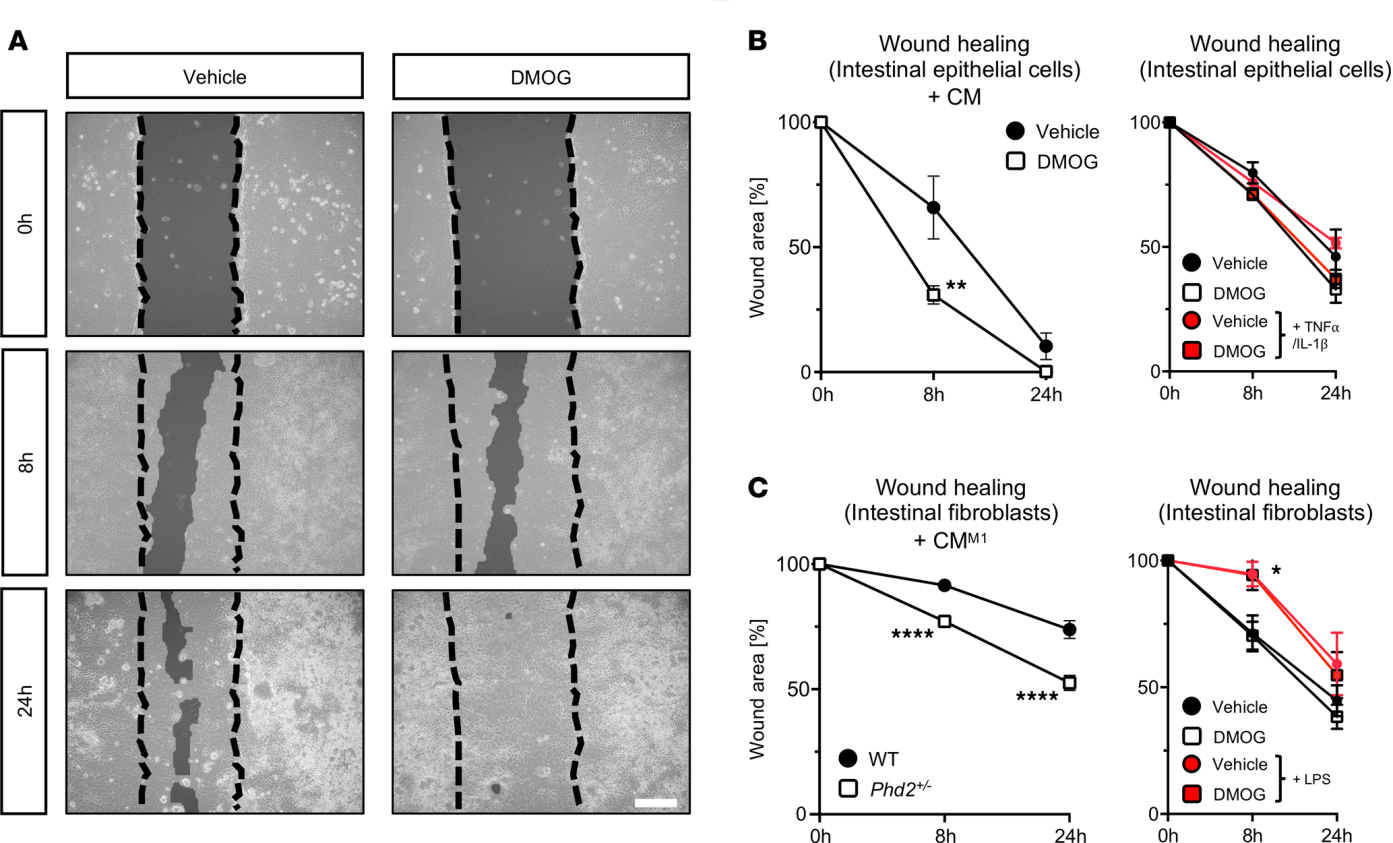
Pharmacologic and genetic inhibition of HIF-prolyl hydroxylases improves wound healing by inducing PHD2-related macrophage polarization. (**A**–**C**) Representative images (**A**) and histomorphometric quantification (**B** and **C**) of scratch assays analyzing wound healing capacity of human intestinal epithelial cells (Caco-2; **A** and **B**) and primary murine intestinal fibroblasts (**C**) treated with conditioned medium (CM; *left graph*) harvested from vehicle- or DMOG-treated (1 mM) human monocyte cell line (THP-1; **A** and **B**, *left graph*) or WT and *Phd2^+/–^* BMDMs after M1 (CM^M1^; *left graph*) polarization with LPS (100 ng/mL). Additionally, wound healing capacity of human intestinal epithelial cells (Caco-2; **B**, *right graph*) and primary murine intestinal fibroblasts (**C**, *right graph*) was quantified after vehicle or DMOG treatment +/– proinflammatory stimulus with TNF-α/IL-1β (Caco-2) or LPS (primary fibroblasts). Scale bar represents 200 μm. Two-way ANOVA with post hoc test. *P < 0.05 vs. control-treated cells; **P < 0.01 vs. CM from vehicle-treated cells; ****P < 0.0001 vs. CM^M1^ from WT. All experiments were performed in biological triplicates.

Finally, we aimed to demonstrate the biological significance of M2 macrophage polarization conferred by PHI or genetic PHD2 haplodeficiency (*Phd2^+/–^*) in the context of anastomotic healing. For this purpose, intestinal epithelial cells and fibroblasts were subjected to in vitro scratch assays applying CM from vehicle- or PHI-treated human macrophages (THP-1 cell line) and CM from primary WT or *Phd2^+/–^* BMDMs, which were initially M1 polarized (CM^M1^; see Methods section). Indeed, closure of scratch wounds in human intestinal epithelial cells (Caco-2 cell line) was significantly accelerated upon addition of CM from PHI-treated human macrophages compared with addition of CM from vehicle control–treated macrophages (Figure 6, A and B, left graphs). Similarly, closure of scratch wounds in primary murine intestinal fibroblasts occurred significantly faster upon addition of CM^M1^ from murine *Phd2^+/–^* macrophages than upon addition of CM^M1^ from WT macrophages (Figure 6C, left graph). Notably, direct DMOG treatment of human intestinal epithelial cells and primary murine intestinal fibroblasts did not alter the closure of scratch wounds compared with vehicle treatment, neither without nor with proinflammatory stimuli (Figure 6, B and C, right graphs).

Collectively, these in vitro findings support the notion that pharmacologic PHD inhibition and genetic PHD2 haplodeficiency induce M2 polarization of macrophages in hypoxic conditions and that this effect augments wound healing properties of intestinal epithelial cells and fibroblasts.

## Discussion

The present study demonstrates that PHD inhibition by DMOG and *Phd2^+/–^* improve healing of colonic anastomoses under both ischemic and septic conditions in 2 relevant preclinical mouse models. Improved healing of colonic anastomoses was accompanied by a marked increase of collagen deposition and reduced cytokine- and chemokine-driven infiltration of leukocytes and macrophages within colonic anastomoses. *Phd2^+/–^*, in particular, elicited a marked reduction of proinflammatory (M1) and induction of immunomodulatory (M2) macrophages, which suggests a “switch” in macrophage polarization within septic colonic anastomoses. Mechanistically, our in vitro findings suggest that PHI- and *Phd2^+/–^*-induced M2 polarization of macrophages enhances wound healing capacities of intestinal epithelial cells and fibroblasts, which overall confers a favorable (pro–wound healing) microenvironment within colonic anastomoses.

Macrophage polarization is crucially involved in wound healing (28). In general, proinflammatory (M1) macrophages are associated with attenuated wound healing, partly due to collagen degradation, whereas immunomodulatory (M2) macrophages are linked to activation of fibroblasts, thereby improving wound healing (9, 28). Our data extend this concept to healing of intestinal anastomoses, showing that PHD inhibition and *Phd2^+/–^* markedly decreased infiltration of M1 macrophages and enhanced numbers of M2 macrophages, overall increasing collagen content within colonic anastomoses. However, the role of PHDs and pharmacologic PHD inhibition during macrophage polarization is more complex, and partly conflicting reports exist in particular on the role of PHD2. PHD inhibition by DMOG, *Phd1^–/–^*, and *Phd2^+/–^* enhance M2 polarization in different murine models of ischemia and inflammation (16, 17, 29). In contrast, myeloid-specific loss of *Phd2* does not convincingly induce M2 polarization of macrophages (30, 31). One possible explanation for these in part conflicting data is that the latter report, which relied on cell culture experiments, did not investigate macrophage polarization under hypoxic conditions (31). The presented data, however, suggest that effects of PHD inhibition and *Phd2^+/–^* on macrophage polarization are rather subtle in normoxia, while hypoxia augments PHI- and *Phd2^+/–^*-mediated M2 polarization. In conclusion, the degree of *Phd2* inhibition, for example *Phd2* deficiency versus *Phd2* haplodeficiency, and the cellular microenvironment, such as normoxia versus hypoxia, seem to be of crucial importance for the role of PHD2 during M2 polarization of macrophages.

Breaking strength of colorectal anastomoses relies on sufficient amounts of collagen, especially during early phases of healing (20, 32). Arginase 1 and TGF-β produced by M2 macrophages are both highly involved in cell proliferation and collagen deposition (33–36). Consistently, we could show that PHI and *Phd2^+/–^* enhanced IL-4–stimulated mRNA expression of *Arg1* and *Tgfb1*, which serves to explain the increased wound healing capacity of intestinal epithelial cells and fibroblasts after treatment with CM from PHI-treated or *Phd2^+/–^* macrophages. On the contrary, chronic hypoxia attenuates fibroblast activity (37). Interestingly in this context, several preclinical studies have shown that hyperbaric oxygen treatment (HBOT) improves healing of intestinal anastomoses and might even induce M2 macrophage polarization; however, these findings could not be translated into clinical practice (3, 38).

One major advantage of pharmacologic HIF upregulation by PHI is that PHI is not only increasing oxygen delivery (like HBOT or erythropoietin) but also decreasing oxygen consumption of resident cells by altering their cellular metabolism (8, 31). In a different context, loss of *Phd1* likewise reduced oxidative stress in liver and peripheral muscle cells in murine models of ischemia, which was partly mediated by pyruvate dehydrogenase kinase–dependent metabolic reprogramming (39–41). Additionally, *Phd2^+/–^* improves tissue oxygenation by, for example, increasing arteriogenesis (recruitment of existing blood vessels) and endothelial lining of newly formed blood vessels without affecting vessel number per se (17, 42). In keeping with these findings, we show here that PHI and *Phd2*^+/–^ had no influence on vessel numbers within tissue from ischemic colonic anastomoses. However, to our surprise, PHI or *Phd2^+/–^* likewise did not significantly change the severity of hypoxia in our model of ischemic colonic anastomoses. One reason for this could be the different microenvironment in which our study was performed. Although the intestine is uniquely adapted to a well-defined “physiological oxygen gradient,” oxygen gradients within metastatic tumors are significantly distorted due to unorganized blood vessel formation or almost absent within peripheral muscles under healthy conditions (17, 42).

Our results encourage the use of nonspecific PHD inhibition to improve healing of critical anastomoses. However, nonspecific PHD inhibition likewise inhibits PHD3, which might provoke adverse effects (43, 44). In particular under septic conditions, genetic loss of *Phd3* has been shown to have detrimental effects on survival in a murine sepsis model (25). Otherwise-beneficial effects of nonspecific PHD inhibition on healing of colonic anastomoses might, therefore, be overshadowed by its inhibitory effects on PHD3 during sepsis. In fact, our data show that while the PHI slightly improved bursting pressure values of septic colonic anastomoses, there was no effect on survival, partly contrasting the protective effects of *Phd2^+/–^*. In addition, our in vitro results do not suggest impaired phagocytotic function of macrophages due to nonspecific PHD inhibition. Nonetheless, it is conceivable that PHD2-specific PHD inhibition would provide a superior safety profile under septic conditions during colorectal surgery.

During the early inflammatory response, leukocytes, such as neutrophils and macrophages, are attracted to the wound to clear it from bacteria that could attenuate healing because of collagen degradation (20). However, a prolonged inflammatory response can also have adverse effects and impair wound healing (36). We show here that PHI and *Phd2^+/–^* markedly attenuated infiltration of proinflammatory cells, most probably by reducing CXCL-1. A recent study showed that DMOG significantly reduced CXCL-1 secretion and, thereby, recruitment of inflammatory cells in a murine model of allergic skin inflammation (45). IL-6 regulates inflammatory cell infiltration and CXCL-1 expression during inflammation (26, 27). Importantly, we could show that PHI and *Phd2^+/–^* reduced IL-6 production in models of ischemic and septic colonic anastomoses. Taken together, PHI-mediated reduction in immune cell infiltration could be due to pharmacologic inhibition of PHD2 and further downstream IL-6/CXCL-1 production of macrophages. However, since in the present study we applied a global knockout mouse model, we cannot rule out that the beneficial effects of *Phd2^+/–^* might also extend to other cell types than macrophages. For example, keratinocyte-specific loss of *Phd2* and topical silencing of PHD2 by siRNA have been shown to accelerate skin wound healing (46, 47). Furthermore, there is increasing evidence that PHDs also hydroxylate alternative non-HIF targets (13). We are thus unable to rule out that genetic or pharmacologic PHD inhibition in the context of anastomotic healing exerts antiinflammatory effects independent of the HIF pathway.

In conclusion, PHD inhibition improves healing of ischemic and septic colonic anastomoses most probably by inhibiting PHD2. Mechanistically, we could show that PHI- and *Phd2^+/–^*-stimulated M2 polarization of macrophages augments wound healing properties of intestinal epithelial cells and fibroblasts, together conferring a favorable (pro–wound healing) microenvironment. Therefore, targeting PHDs by PHI to prevent AL might represent a promising treatment option, particularly in patients with critically perfused colorectal anastomosis. In patients with abdominal sepsis, selective inhibition of PHD2 might prove beneficial compared with nonselective PHD inhibition.

## Methods

### Mouse model of colonic anastomoses.

Up to 4 animals were kept under specific pathogen–free conditions in single cages, with food and water given ad libitum with a 12-hour light/12-hour dark cycle. Power analysis was performed to determine the number of animals required per group. Female and male WT mice aged 10 to 15 weeks were randomly assigned to different treatment groups and operated on by the same surgeon. In defined experiments (“pretreatment”), WT mice were (pre-)treated with daily i.p. injections of vehicle (Aqua; MilliporeSigma) or DMOG, 100 μg/g BW (18, 48) (PubChem CID: 560326; Biomol), for the whole duration of the experiment (day –3 to day 3). The generation of globally *Phd1*-deficient (*Phd1^–/–^*), *Phd2*-haplodeficient (*Phd2^+/–^*), and *Phd3*-deficient (*Phd3*^–/–^) mice has previously been described (25, 39).

Mice underwent general anesthesia and afterward midline laparotomy colonic anastomoses were created on day 0. To induce ischemia-mediated impairment of anastomotic healing, critical colonic blood vessels were ligated and dissected before creation of anastomoses (20). For experiments analyzing sepsis-inflicted impairment of anastomotic healing, mice received a single i.p. injection of LPS (*Escherichia coli* O111:B4; L2630; MilliporeSigma) 18 hours before creation of colonic anastomoses to induce abdominal sepsis (49). On day 3, colonic anastomoses were harvested and analyzed for indirect signs of AL (gross morphology) and bursting pressure as previously described (20, 49). Briefly, after excision and cannulation of the colonic anastomoses, bursting pressure was continuously recorded upon stepwise filling of the colonic anastomoses with air. The pressure at leakage was recorded as bursting pressure. Colonic anastomoses were further subjected to histology or mRNA and protein expression analyses as outlined below. Details on the surgical procedure to create colonic anastomoses under baseline conditions, ischemia and sepsis, and other related procedures, such as bursting pressure analysis and daily measurement of DAI (Supplemental Table 2 and ref. 25), are described in the Supplemental Methods. Blood samples were taken on day 3 to determine red blood cell count, hemoglobin values, and hematocrit values of vehicle- and DMOG-treated animals (Supplemental Table 1).

### Detection of HIF-1α protein levels.

For quantification of HIF-1α protein levels, we used a commercially available ELISA kit (Abcam) detecting DNA binding activity as described before (50). HIF-1α levels were assessed in tissue lysates from anastomotic colonic biopsies harvested from vehicle- and DMOG-treated animals. Absorbance was measured at 450 nm. All experiments were performed in accordance with the instructions provided by the manufacturer and in triplicates.

### Histology and immunohistochemistry.

Paraffin-embedded tissue was sectioned at 5 μm thickness, dewaxed, and rehydrated in xylene and graded ethanol series. Tissue was routinely stained with hematoxylin and eosin to identify regions of interest within colonic anastomoses (Merck; see Supplemental Figure 2A). MTG (Merck) staining was conducted to dye collagen fibers according to manufacturer’s instructions.

For immunohistochemistry, antigens were retrieved with Target Retrieval Solution (Dako), blocked with serum from the same species the secondary antibody was raised in (Vector Laboratories), and incubated overnight with the following primary antibodies: CD45 (1:100; 553076, BD Pharmingen), F4/80 (1:100; MCA497, Bio-Rad), αSMA (1:100; ab5694, Abcam), CD31 (1:100; ab28364, Abcam), arginase 1 (1:200; R30878, NSJ Bioreagents), iNOS (1:100; ab15323, Abcam), and CXCR2 (1:100; ab14935, Abcam). The following day, appropriate secondary antibody (BA-4001 and BA-1100, Vector Laboratories) was added and amplified with TSA Indirect (PerkinElmer), before DAB labeling (Dako). Quantification of positively stained areas was carried out by 2 independent, blinded investigators, on 10 HPFs using an Axiostar Plus Microscope (Carl Zeiss) and ImageJ software (NIH).

### Hypoxia labeling.

Detection of hypoxia by pimonidazole immunostaining has been previously described (51). Briefly, pimonidazole (60 mg/kg BW; Hypoxyprobe) was administered to the peritoneal cavity 60 minutes prior to harvesting of colonic anastomoses. Paraffin-embedded tissue sections were dewaxed and rehydrated as described above. After blocking, slides were incubated with the primary antibody (1:100; rabbit anti-pimonidazole antibody, Pab2627, Hypoxyprobe) overnight. The next day, slides were washed and incubated with the appropriate secondary antibody before DAB labeling. Characteristic staining patterns, revealing intense pimonidazole staining close to the anoxic gut lumen, and weak staining at basal mucosal layers, confirmed successful hypoxia labeling (compare Supplemental Figure 2B). Quantification of positively stained cells was carried out as described above.

### Quantitative RT-PCR.

Total RNA was isolated using RNeasy Mini Kit (Qiagen) or TRIzol (TRI Reagent; MilliporeSigma) and transcribed into cDNA with Improm-II-Reverse Transcription System (Promega). Quantitative PCR was performed using the LightCycler 480 (Roche) or the Applied Biosystems QuantStudio 7 Flex System (Thermo Fisher Scientific) and fluorescence-based chemistry assays (SYBR Green I dye) with specific primers (Supplemental Table 3). Transcript levels were calculated relative to the appropriate housekeeping gene as indicated.

### Detection of protein levels.

Total protein lysates from ischemic colonic anastomotic tissue were prepared using T-PER Tissue Protein Extraction Reagent (Thermo Fisher Scientific) and Tissue Lyser II (Qiagen). Lysates were then analyzed with a Mouse XL Cytokine Array (ARY028, R&D Systems, Bio-Techne). Chemiluminescence was detected with Fusion SL2-3500.WL (Vilber Lourmat). Densitometric analysis of dots of each cytokine was performed with ImageJ. Background signal was subtracted, and expression of each cytokine was normalized to its own positive control.

In addition, IL-6 protein levels were determined in whole tissue lysates from septic colonic anastomoses using a commercially available ELISA (M6000B, R&D Systems, Bio-Techne). In CM from LPS-polarized proinflammatory (M1) BMDMs, IL-6 protein and CXCL-1 were also analyzed by ELISA (IL-6: M6000B, R&D Systems, Bio-Techne; CXCL-1: MKC00B, R&D Systems, Bio-Techne). Absorbance was measured at 450 nm. Further details on culture conditions are outlined below. All experiments were performed in triplicate.

### Cell culture experiments.

J774.A1 (murine monocyte cell line), THP-1 (human monocyte cell line), and Caco-2 cells (human intestinal epithelial cell line) were purchased from ATCC (LGC Standards) and cultured in DMEM (J774.A1 and Caco-2; MilliporeSigma) or RPMI-1614 (THP-1; MilliporeSigma) with 10% FCS and 1% penicillin/streptomycin. Cells were cultured at 5% CO_2_ and ambient oxygen concentrations (21% O_2_) at 37°C unless otherwise indicated.

For polarization experiments, J774.A1 cells were differentiated into macrophages with 200 nM phorbol myristate acetate (PMA; MilliporeSigma) for 48 hours and subsequently subjected to 24 hours’ normoxia (21% O_2_) or hypoxia (0.75% O_2_). Cells were additionally treated with 100 ng/mL LPS (MilliporeSigma) to induce proinflammatory (M1) macrophages, or 10 ng/mL IL-4 (MilliporeSigma) to induce immunomodulatory (M2) macrophages, for 18 hours. Vehicle (DMSO; MilliporeSigma) or DMOG treatment (1 mM; Biomol) of cells was performed simultaneously as indicated. BMDMs were isolated and identified as previously reported (51). Briefly, femur and tibia were flushed with PBS, and cells were differentiated in RPMI-1640 (MilliporeSigma) with 10% FCS, 1% penicillin/streptomycin, 2 mM l-glutamine, and 10 ng/mL mM-CSF (R&D Systems, Bio-Techne) for 7 days prior to experiments. BMDMs of WT and *Phd2*-haplodeficient mice were treated analogous to J774.A1 cells with LPS (M1) and IL-4 (M2) under normoxia and hypoxia. Cells were lysed for further analysis of mRNA expression at the end of the experiment. To measure phagocytosis, cells were incubated with pHrodo Red E. coli BioParticles Conjugate (Thermo Fisher Scientific). Fluorescence intensity was assessed at 535 nm/595 nm.

Primary intestinal fibroblasts were isolated as previously described (51, 52). Briefly, intestinal tissue was minced and digested with collagenase (MilliporeSigma). Primary intestinal fibroblasts were cultured in Eagle’s minimum essential medium (MilliporeSigma) with 15% FCS, 1% penicillin/streptomycin, and 2 mM l-glutamine (MilliporeSigma). To determine cell identity, cells were grown on coverslips, fixed, and labeled with a primary antibody labeling fibroblast-specific protein 1 (1:100; ab27957, Abcam) and an FITC-labeled secondary antibody (1:1000; ab6717, MilliporeSigma) (Supplemental Figure 3A and ref. 51).

Wound healing capacity was analyzed with monolayer scratch assays using CM or direct treatment with vehicle or DMOG without and with proinflammatory stimuli. Since Caco-2 cells do not express TLR4, Caco-2 cells were treated with TNF-α/IL-1β (both 10 ng/mL) for 18 hours to induce inflammation. In primary murine fibroblasts, inflammation was induced by LPS treatment (100 ng/mL for 18 hours). To generate CM, human monocytes (THP-1 cell line) were first differentiated into macrophages with 320 nM PMA (MilliporeSigma) for 3 days. After vehicle (DMSO) or DMOG (1 mM) treatment for 24 hours, CM was collected, filtered (0.22 μm sterile filter unit; Millex-GP, Merck), and stored at –80°C for later experiments. For generation of CM^M1^, primary WT and *Phd2^+/–^* BMDMs were polarized as indicated above, and medium was collected after 48 hours analogous to CM from THP-2 cells. Similarly treated CM, which was collected from culture experiments without cells, served as negative controls (CM_no_ _cells_) and did not alter the wound healing capacity of Caco-2 cells per se (Supplemental Figure 3B). Next, Caco-2 cells and primary intestinal fibroblasts were grown to confluence and scratched using a 200 μL pipette as previously described (53). After washing, Caco-2 cells or primary intestinal fibroblasts were treated with CM from vehicle- or DMOG-treated human macrophages (THP-1), or with CM^M1^ from WT and *Phd2^+/–^* BMDMs, respectively, in a 1:1 ratio (CM/fresh medium). Documentation of gap closure by microscopy was performed after 0 hours, 8 hours, and 24 hours. Open wound areas were measured using TScratch (53) in a blinded fashion. For each well 5 images at original magnification ×40 were taken and assessed. Means for each well, as well as differences over time, were calculated. All cell culture experiments were repeated at least 3 times. Presented graphs depict pooled data from individual experiments.

### Flow cytometry.

To analyze protein expression of cell surface markers of macrophage polarization, flow cytometry was performed. Cells were stained with different fluorescence antibodies (see Supplemental Table 4) and finally analyzed according to the following protocol. Two million cells (THP-1 cells) were grown in 6-well plates, and monocyte differentiation and macrophage polarization were induced as described above. For analysis, cells were gently detached, and the cell suspension was transferred into an ice-cold 15 mL Falcon tube (Greiner Bio-One International), which was tightly sealed. The following protocol was performed within the gas chamber unless otherwise indicated. Cell suspensions were centrifuged at 1000*g* for 5 minutes and washed with ice-cold PBS to prevent internalization of surface proteins. The supernatant was removed and ice-cold PBS was added to subsequently count the cells by trypan blue staining. To guarantee equal staining, 400,000 cells were resuspended in 100 μL of FACS buffer consisting of Dulbecco’s PBS (Gibco, Thermo Fisher Scientific) and 10% FBS (MilliporeSigma). Antigen blocking was performed to prevent nonspecific binding of the primary antibody binding by adding 1 μL of diluted flow cytometry blocking reagent (1:10; BD Pharmingen; 564219). The suspension was gently mixed and incubated at room temperature for 10 minutes. Without washing, diluted antibodies were added to the cell suspension and mixed by vortexing. The following steps were performed outside the gas chambers. The tubes were placed in the dark from that point on and incubated at 8°C for 10 minutes. Staining was stopped by adding 1 mL of FACS buffer and centrifugation (1000*g* for 5 minutes). The supernatant was discarded and the cell pellet was washed using ice-cold PBS. Finally, 200 μL of FACS buffer and 1 μL of the viability dye DRAQ7 (Biostatus Ltd.) were added.

After fluorescence staining, scatter characteristics and fluorescence signals were detected using the CytoFLEX LX (Beckman Coulter) at “slow” flow rate (14 μL/min, 10 μm core size). Compensation for multicolor flow cytometry was performed before experimental analysis. Single stained antibodies, consisting of a single antibody mixed with macrophages, were used for compensation (Supplemental Figure 10A). Fluorescence minus one controls were used for defining the gates with a cutoff of 0.1%. Viable single cells were gated by scatter characteristics and negative selection of DRAQ7-positive cell population utilizing a predefined gaiting strategy (Supplemental Figure 10B). Subsequently, a minimum of 10,000 single cells were analyzed, and the median fluorescence intensity and the number of positively stained cells were recorded to quantify protein expression. FCS Express 7 software (De Novo Software) was applied to plot two-dimensional graphs and calculate median values. Statistical analysis was performed as described below.

### Statistics.

Statistical analysis was carried out with GraphPad Prism version 7 (GraphPad Software). Differences in frequencies of categorical data were determined by χ^2^ test. Continuous data sets from 2 groups or more were analyzed by Student’s *t* test or ANOVA test with appropriate post hoc test, respectively. Student’s *t* tests were 2-tailed and 1-way ANOVA was performed if not otherwise indicated in the figure legends. The Kaplan-Meier method was applied to estimate animal survival rates, and survival curves were compared by log-rank test. Data sets from the antibody array were analyzed by multiple *t* tests with correction for multiple testing by Holm-Šidák method. All data sets were tested for normal distribution. If data were not normally distributed, appropriate nonparametric tests were performed as indicated in the figure legends. Data are given as mean ± SEM. A *P* value less than 0.05 was considered significant.

### Study approval.

Animal experiments were approved by the local animal welfare committee (Regierungspräsidium Karlsruhe; G-208/13) and performed in accordance with the NIH *Guide for the Care and Use of Laboratory Animals* (NIH Publication No. 8023, revised 1978, National Academies Press) (see also ARRIVE checklist).

## Author contributions

MJS, CTT, and MS designed the study. MJS, GK, JW, ASR, PR, VMO, CT, and UK conducted experiments. MJS, GK, JW, ASR, PR, VMO, CT, UK, JMH, J Kugler, AB, J Klose, MB, and TS conducted further data acquisition and analysis. MJS and MS drafted the manuscript and prepared the figures. All authors critically revised and approved the final manuscript version.

## Supplementary Material

Supplemental data

## Version 1 03/30/2021

In-Press Preview

## Version 2 04/22/2021

Electronic publication
