# Supplementary material for: Inhibition of HIF-prolyl hydroxylases improves healing of intestinal anastomoses
Source: JCI Insight. 2021 Mar 30;6(8):e139191. doi: 10.1172/jci.insight.139191 (PMC8119215; doi:10.1172/jci.insight.139191)
Supplement: Supplemental data [file jciinsight-6-139191-s007.pdf]

INHIBITION OF HIF-PROLYL HYDROXYLASES IMPROVES HEALING OF INTESTINAL ANASTOMOSES

Moritz J. Strowitzki[1,2], Gwendolyn Kimmer[1], Julian Wehrmann[1], Alina S. Ritter[1], Praveen Radhakrishnan[1], Vanessa M. Opitz[1], Christopher Tuffs[1], Marvin Biller[1], Julia Kugler[2], Ulrich Keppler[1,3], Jonathan M. Harnoss[1], Johannes Klose[1], Thomas Schmidt[1], Alfonso Blanco[4], Cormac T. Taylor[2] and Martin Schneider[1]

(1) Department of General, Visceral and Transplantation Surgery, Heidelberg University, Heidelberg, Germany.

(2) School of Medicine and Conway Institute of Biomolecular & Biomedical Research, University College Dublin, Dublin, Ireland.

(3) Department of Anaesthesiology, Heidelberg University, Heidelberg, Germany.

(4) Flow Cytometry Core Technology. Conway Institute of Biomolecular & Biomedical Research, University College Dublin, Dublin, Ireland.

SUPPLEMENTARY MATERIAL

SUPPLEMENTARY NOTES

DETAILED DESCRIPTION OF THE MOUSE MODEL OF COLONIC ANASTOMOSES

The creation of colonic anastomoses in mice has been described elsewhere before (1, 2). Under baseline conditions, mice were anaesthetized with isoflurane (Isothesia, Henry Schein, Hamburg, Germany) on day 0 and after midline laparotomy colonic anastomoses were created by sharp dissection of the distal colon, which was followed by re-anastomization of the two colon segments using 12 stiches and 7-0 Prolene (Ethicon, Norderstedt, Germany) as suture material.

To induce ischemia-inflicted impairment of anastomotic healing, colonic anastomose were additionally challenged by ligating and dissecting critical blood vessels 1cm proximal and distal of the prospective anastomotic suture line creation of the colonic anastomoses as described previously (Figure 1*A*) (2). In sham-treated controls, critical vessels were carefully mobilized but neither ligated nor dissected. Colonic anastomoses were created as described above. For sepsis-inflicted impairment of anastomotic healing, colonic anastomoses were challenged by inducing abdominal sepsis 18h before creation of the anastomoses (Figure 3*A* and *B*) (1). Abdominal sepsis was induced by intraperitoneal injection of 0.1 or 0.5mg/kg BW of lipopolysaccharides (LPS; Escherichia coli O111:B4; L2630; Sigma-Aldrich) as indicated. Control animals were treated with equal amounts of vehicle solution (Aqua; Sigma-Aldrich). Note, that after optimization only high dosage of LPS (0.5mg/kg BW) was used to induce septic colonic anastomoses. Afterwards perioperative outcome was analysed by daily measurements of semi-quantitative disease activity index (DAI), which included analysis of “posture and appearance”, “alertness” and “ruffled fur” (Supp. Table 2) and recording of post-operative survival until day 3 (Figure 3*B*) (3).

In all animals, the peritoneum and skin were closed with 6-0 PDS II (Ethicon). For postoperative pain management all animals received a single subcutaneous injection of buprenorphine (0.05mg/kg BW). On day 3 all animals were re-anaesthetized with isoflurane and analysed for indirect signs of anastomotic leakage, such as adhesion and abscess formation, and gross structural defects (“anastomotic insufficiencies”) (2). Afterwards colonic anastomoses were

harvested for bursting pressure analysis as described elsewhere (1). Briefly, colonic anastomoses were carefully and sharply excised 1.5cm proximal and distal from the created anastomoses. Gross adhesions and abscess formation were kept in place, as removal could have impaired structural integrity of the anastomotic segment. While distal ends of the anastomoses were ligated, the proximal end was tightly cannulated. The bursting pressure was continuously recorded on a pressure transducer during stepwise filling of the colonic anastomoses with air while placing the colon under water. The pressure at leakage was recorded as the "bursting pressure". After bursting pressure analysis colonic anastomoses were randomly (1:1 ratio) assigned for further histological or mRNA and protein expression analysis. All animals were sacrificed by cervical dislocation at the end of the experiment.

WESTERN BLOTTING

Whole-cell lysates were prepared in radioimmunoprecipitation assay buffer (50mM Tris-Cl pH 8.0, 150mM NaCl, 0.1% SDS, 0.5% sodium deoxycholate, 1% Triton X-100, 1x Protease Inhibitor Cocktail). Cell suspensions were triturated 10-12 times through a 26G needle and incubated on ice with constant agitation. Lysates were centrifuged and protein content of lysates was quantified and normalized using BCA assay (Pierce™; Thermo Fisher Scientific). Western blot analysis was performed as previously described (4) utilizing the following antibodies: HIF-1α (1:500; BD Biosciences; 61095), β-Actin (1:10000; Sigma-Aldrich/Merck, A5441), anti-rabbit IgG HRP-linked (1:1000; Cell Signaling Technology; 7074) for HIF-1α, and anti-mouse IgG, HRP-linked (1:10000; Cell Signaling Technology; 7076) for β-Actin.

REFERENCES

1. Diller R et al. ATIII Attenuates Endotoxemia Induced Healing Impairment in the Colon. *J. Surg. Res.* 2009;157(1):4–13.
2. Shogan BD et al. Collagen degradation and MMP9 activation by Enterococcus faecalis contribute to intestinal anastomotic leak. *Sci. Transl. Med.* 2015;7(286):286ra68-286ra68.
3. Kiss J et al. Loss of the Oxygen Sensor PHD3 Enhances the Innate Immune Response to Abdominal Sepsis. *J. Immunol.* 2012;189(4):1955–1965.
4. Strowitzki MJ et al. High hepatic expression of PDK4 improves survival upon multimodal treatment of colorectal liver metastases. *Br. J. Cancer* 2019;120(7):675–688.

SUPPLEMENTARY TABLES

Supp. Table 1 Blood sample analysis of vehicle- and DMOG-treated animals during healing of intestinal anastomoses under ischemic conditions on day 3.

	**Vehicle (N = 8)**		**DMOG (N = 10)**		
	Mean	SEM	Mean	SEM	*P-value*
RBC [/pl]	7.03	0.21	7.70	0.21	0.036
Hemoglobin [g/dl]	11.53	0.26	13.4	0.26	0.000
Hematocrit [l/l]	0.33	0.01	0.36	0.01	0.044

DMOG dimethyloxalylglycine; *RBC* red blood cell count; *SEM* standard error of the mean.

Supp. Table 2 Parameters included in disease activity index (DAI).

<table>
<tr><td colspan="6">DAI</td></tr>
<tr><td></td><td>0 = none present</td><td>1 = mild</td><td>2 = moderate</td><td>3 = severe</td><td>score</td></tr>
<tr><td rowspan="3">posture + appearance</td><td>normal posture</td><td>hunched</td><td>hunched</td><td>hunched</td><td rowspan="3">0 - 3</td></tr>
<tr><td></td><td>no bloating</td><td>bloated abdomen</td><td>markedly bloated abdomen</td></tr>
<tr><td></td><td></td><td>sunken eyes</td><td>conjunctival injection</td></tr>
<tr><td rowspan="3">alertness</td><td>active</td><td>alert</td><td>depressed level of alertness</td><td>markedly (or absent) depressed level of alertness</td><td rowspan="3">0 - 3</td></tr>
<tr><td>interactive in environment</td><td>occasional interest in environment</td><td>little interest in environment</td><td>no interest in environment</td></tr>
<tr><td></td><td>moves freely</td><td>moves difficultly</td><td>no movement</td></tr>
<tr><td>fur</td><td>well-groomed</td><td>mild piloerection</td><td>moderate piloerection (> 20 %)</td><td>marked piloerection (> 60 %)</td><td>0 - 3</td></tr>
<tr><td></td><td></td><td></td><td></td><td>total score</td><td>0 - 9</td></tr>
</table>

Supp. Table 3 mRNA primer sequences for q-RT-PCR.

Gene	Synonym	Forward Sequence	Reverse Sequence
Acta2	αSMA	5'CTTTTCCATGTCGTCCCAGT3'	5'AATGGCTCTGGGCTCTGTAA3'
Actb	β-Actin	5'GCTGTATTCCCCTCCATCGT3'	5'AGGTGTGGTGCCAGATCTTC3'
Arg1	Arginase-1	5'CATCGTGTACATTGGCTTGC3'	5'CTTCCATCACCTTGCCAATC3'
Cxcl1	CXCL-1	5'TCTCCGTTACTTGGGGACAC3'	5'GCTGGGATTCACCTCAAGAA3'
Slc2a1	Glut1	5'GCGGGAGACGCATAGTTACA3'	5'TCACCTTCTTGCTGCTGGG3'
GUSB	GUSb	5'GATCCACCTCTGATGTTCACTG3'	5'TTTATTCCCCAGCACTCTCG3'
Hk1	HK1	5'TCTACAAACTCCATCCACACTTCT3'	5'GGAAACACCACTCCGACTTTTG3'
Il1b	IL-1β	5'TTGACGGACCCCAAAAGATG3'	5'AGAAGGTGCTCATGTCCTCA3'
Il6	IL-6	5'ACAAAGCCAGAGTCCTTCAGAG3'	5'TCCTAGCCACTCCTTCTGTG3'
Il10	IL-10	5'TGAATTCCCTGGGTGAGAAG3'	5'CATGGCCTTGTAGACACCTTG3'
Nos2	iNOS	5'GGAAGAAATGCAGGAGATGG3'	5'AGCTGCTTTTGCAGGATGTC3'
Serpine1	PAI-1	5'TAGCACAGGCACTGCAAAAG3'	5'GTTGTGCCGAACCACAAAG3'
Tgfb1	TGF-β1	5'CCACCTGCAAGACCATCGAC3'	5'CTGGCGAGCCTTAGTTTGGAC3'
Tlr2	TLR2	5'GCAAACGCTGTTCTGCTCAG3'	5'AGGCGTCTCCCTCTATTGTATT3'

αSMA alpha smooth muscle actin; *EGLN* egl-9 family hypoxia inducible factor; *GUSb* glucuronidase beta; *CXCL-1* C-X-C motif chemokine ligand 1; *iNOS* inducible nitric oxide synthase; *IL-10* interleukin 10; *PHD* hypoxia-inducible factor (HIF) prolyl-hydroxylase; *TGF-β1* transforming growth factor beta 1.

Supp. Table 4 Fluorescence antibodies and dyes for flow cytometry.

Target	Target cell/marker	Reactivity	Conjugation	Monoclonal (m)/polyclonal (p)	Dilution	Company	Product code	Instruments	Laser	Filters
Anti-CD206	Macrophages	Human	APC	m	1:100	BD Biosciences	321110	CytoFLEX LX	Red (638nm)	6610/10 BP
Anti-HLA-DR	Macrophages	Human	FITC	m	1:100	BD Biosciences	BDB555558	CytoFLEX LX	Blue (488nm)	525/40 BP
DRAQ7	Cell viability	All species	Unconjugated	n/a	1:200	BioLegend	424001	CytoFLEX LX	Red (638nm)	763/43 BP

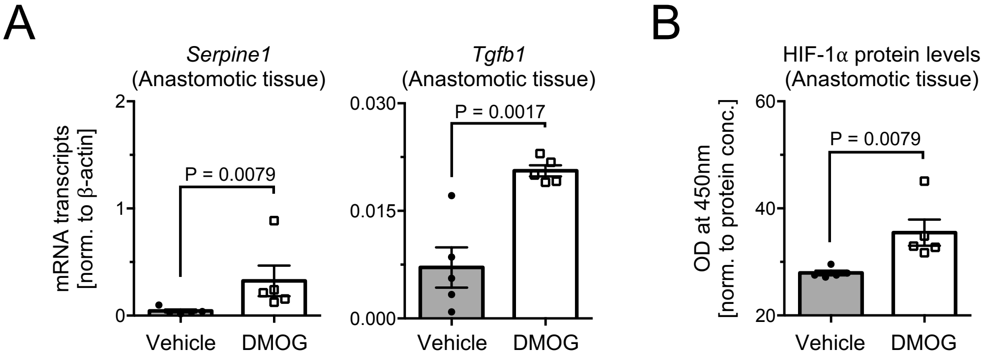

Supp. Figure 1. ***(A)*** Real-time PCR analysis of *Serpine1* and *Tgfb1* mRNA expression within ischemic anastomoses (whole tissue) from vehicle- and DMOG-treated mice [n = 5 biological replicates; Mann-Whitney U (*Serpine1* mRNA levels) or Student's t test (*Tgfb1* mRNA levels)]. ***(B)*** ELISA-based detection of HIF-1α protein levels in whole tissue lysates within ischemic anastomoses (whole tissue) from vehicle- and DMOG-treated mice (n = 5 biological replicates; Mann-Whitney U test).

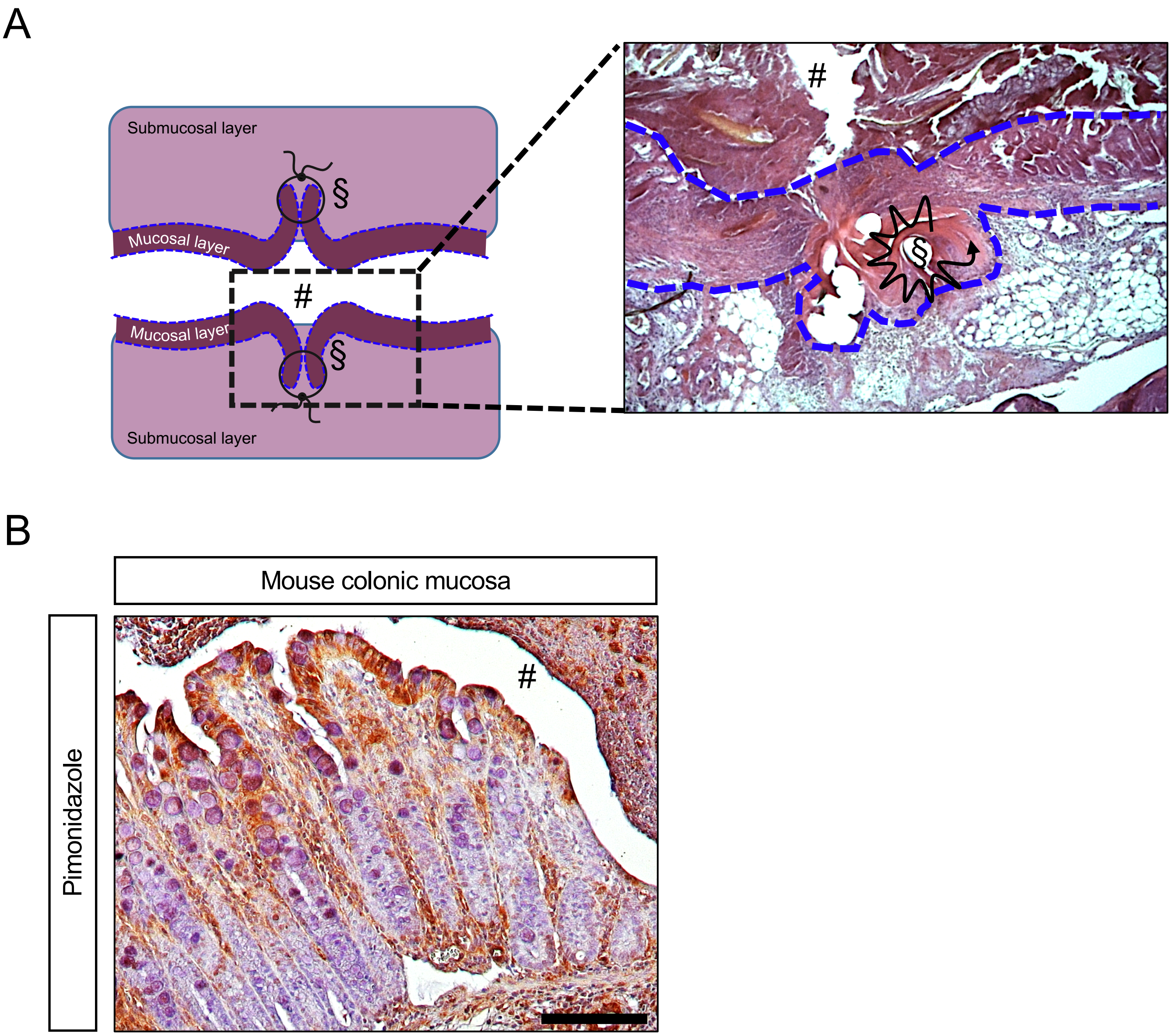

Supp. Figure 2. ***(A)*** *Microscopic appearance of colonic anastomoses*. Schematic drawing (l*eft*) and representative haematoxylin and eosin staining (*right*) of a colonic anastomosis, depicting gut lumen (#) and former position of suture material (§). Dashed blue lines indicate the mucosal layer. The black arrow marks the region of interest, which was further analysed by immunohistochemistry. ***(B)*** Representative immunocytochemistry of pimonidazole in mouse colonic mucosa, showing characteristic hypoxia patterns with high pimonidazole staining indicating anoxic regions close to the gut lumen (#) and low or absent staining in basal regions (scale bar represents 200µm).

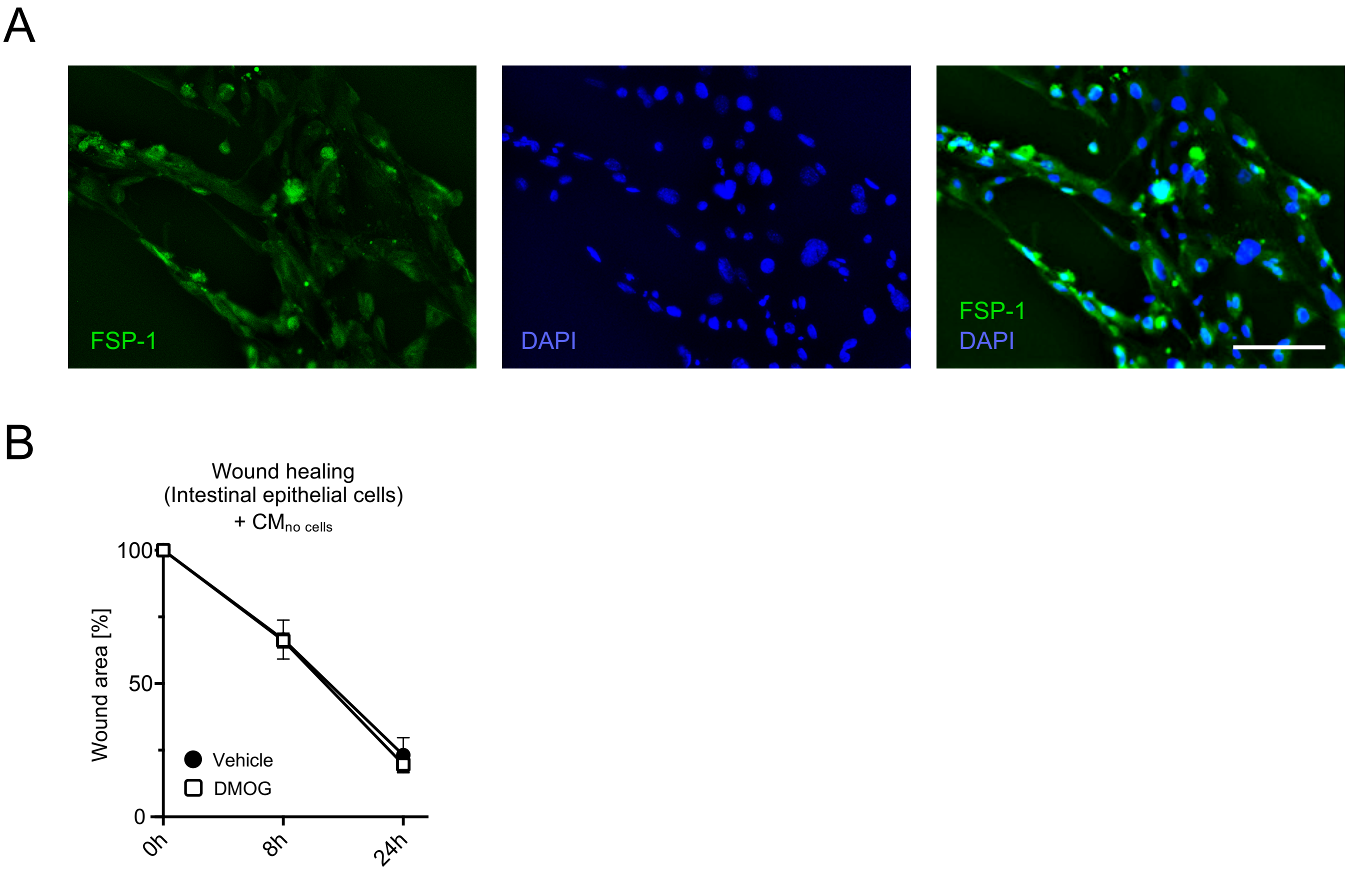

Supp. Figure 3. ***(A)*** Representative immunocytochemistry for fibroblast-specific protein 1 (FSP-1, green) and DAPI (cell nuclei, blue) to confirm primary intestinal fibroblasts (scale bar represents 100 µm). ***(B)*** Histomorphometric quantification of scratch assays analyzing wound healing capacity of human intestinal epithelial cells (Caco-2 cells) treated with conditioned medium generated from cell culture medium without cells ($CM_{no\ cells}$), however, containing similar amounts of vehicle or DMOG (1mM) and LPS (100ng/ml). Experiments in *b* thus represent the negative control for experiments with CM generated as described in the methods. All experiments were performed in biological triplicates.

A

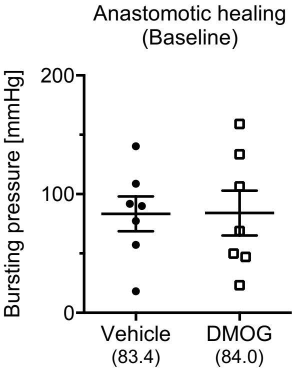

B

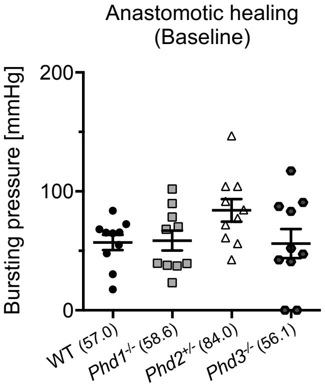

C

	WT n/n (%)	*Phd1$^{-/-}$* n/n (%)	*Phd2$^{+/-}$* n/n (%)	*Phd3$^{-/-}$* n/n (%)
Adhesion	8/10 (80)	8/10 (80)	4/10 (40)	10/10* (100)
Abscess	1/10 (10)	1/10 (10)	1/10 (10)	6/10* (60)
Anastomotic insufficiency	0/10 (0)	0/10 (0)	0/10 (0)	3/10* (30)

D

	WT n/n (%)	*Phd1$^{-/-}$* n/n (%)	*Phd2$^{+/-}$* n/n (%)
Adhesion	11/11 (100)	8/10 (80)	6/11* (55)
Abscess	10/11 (91)	4/10* (40)	5/11* (45)

Supp. Figure 4. ***(A - C)*** *Effects of pharmacologic and genetic inhibition of HIF-prolyl hydroxylases on healing of colonic anastomoses under baseline conditions*. ***(A + B)*** Bursting pressure analysis of healthy colonic anastomoses on day 3 under baseline conditions harvested from vehicle- or DMOG-treated mice (*A;* n = 7 per group) or from WT, *Phd1$^{-/-}$*, and *Phd2$^{+/-}$* mice (*B;* n = 10 per group). ***(C)*** Quantification of indirect signs of anastomotic leakage, such as adhesion or abscess formation, or direct signs of anastomotic insufficiency, such as structural defects, in healthy colonic anastomoses of WT, *Phd1$^{-/-}$*, and *Phd2$^{+/-}$* mice, revealing markedly increased adhesions, abscesses and structural defects only in *Phd3$^{-/-}$* mice (n = 10 animals per group; *P < 0.05 by Chi-square test). ***(D)*** *Effects of genetic inhibition of HIF-prolyl hydroxylases on healing of colonic anastomoses under ischemic conditions*. Macroscopic quantification of indirect signs, such as adhesion or abscess formation, of anastomotic leakage in ischemic colonic anastomoses of WT, *Phd1$^{-/-}$*, and *Phd2$^{+/-}$* mice, showing less adhesions and abscesses only in *Phd2$^{+/-}$* mice (n = 10-11 animals per group; *P < 0.05 by chi-square test).

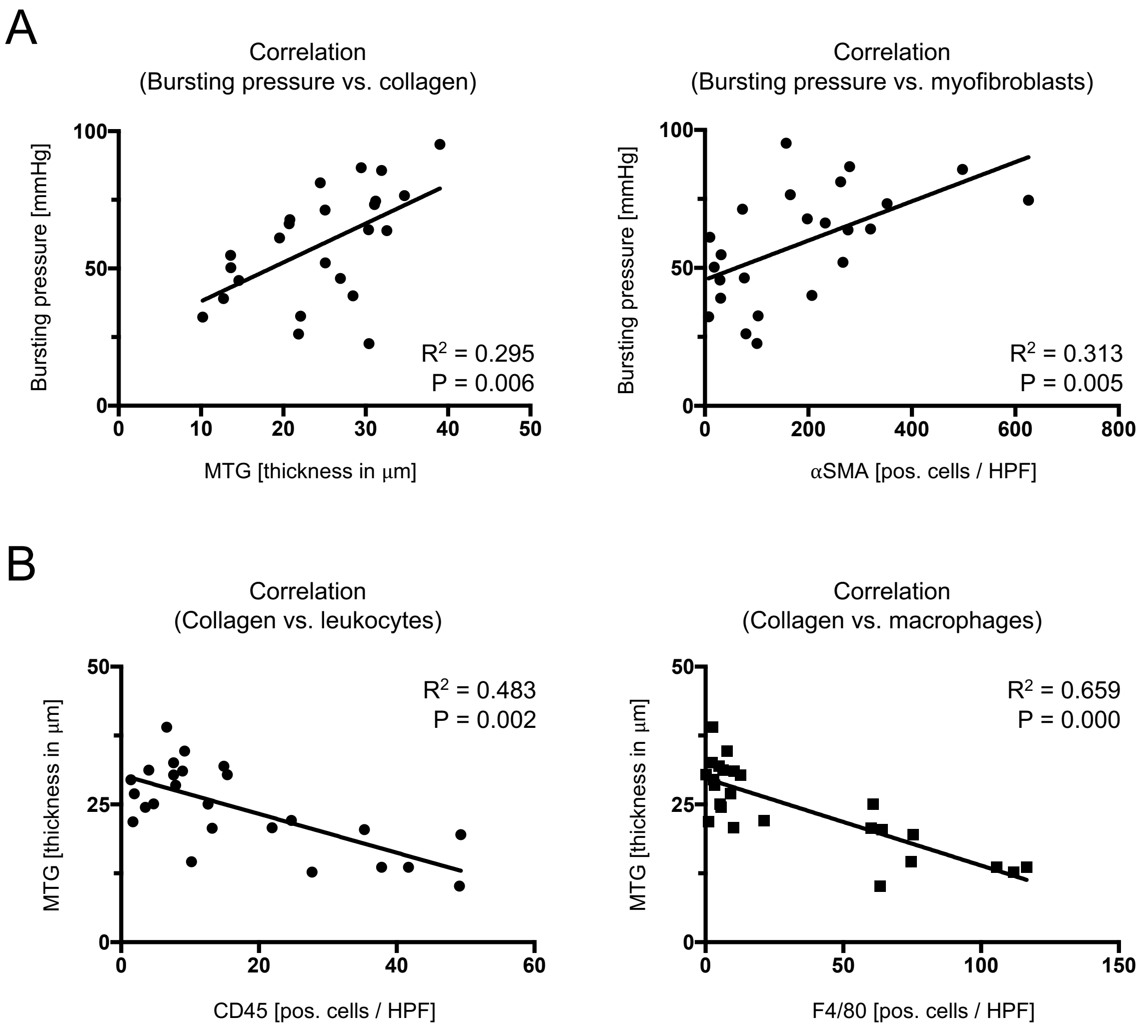

Supp. Figure 5. ***(A + B)*** Correlation analysis of individual bursting pressure values with either collagen capsule thickness (*A left graph*) or αSMA-positive cells – a marker for myofibroblast infiltration (*A right graph*) and correlation of individual capsule thickness with either CD45- (*B left graph*) or F4/80-positve cells (*B right graph*) – markers for leukocyte and macrophage infiltration, respectively (n = 25 animals; correlation was calculated by "Best-fit" logistic regression, significance level and R^2 are given as Pearson's correlation coefficients).

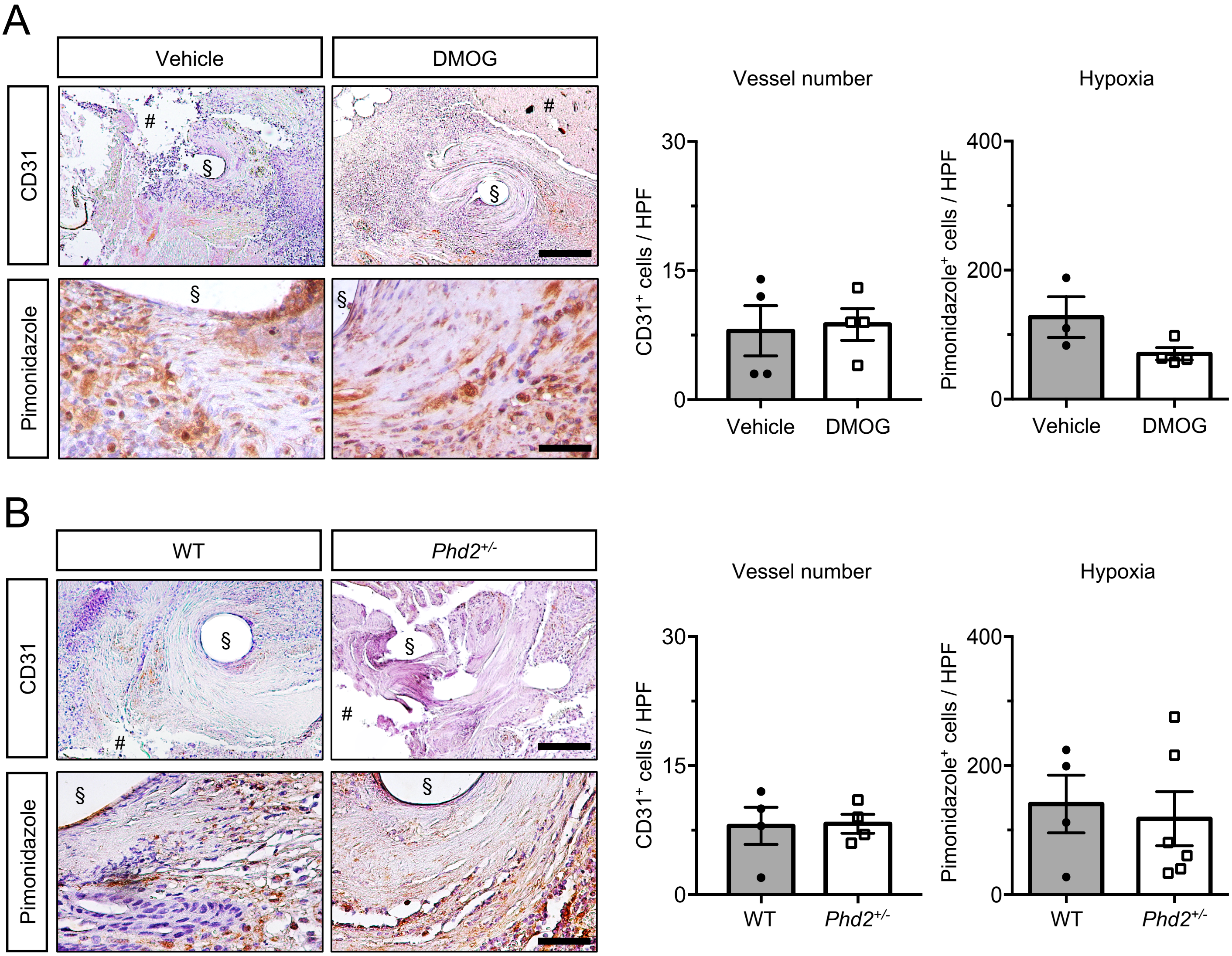

Supp. Figure 6. *Effects of pharmacologic or genetic HIF-prolyl hydroxylase inhibition on vascularization and hypoxia within ischemic colonic anastomoses.* ***(A + B)*** Representative images (*left*) and histomorphometric quantification (*right*) of CD31 (*left upper panels*) and pimonidazole (*left lower panels*) immunostaining of vehicle- and DMOG-treated ischemic colonic anastomoses (*A*) or anastomosis from WT and *Phd2*$^{+/-}$ mice (*B*). Note, that neither DMOG nor *Phd2*-haplodeficiency affected vessel number or the number of ischemic cells within ischemic colonic anastomoses. [n = 3-4 animals per group; scale bars in upper and lower panels (in *A* and *B*) represent 200µm and 100µm, respectively; § and # indicate positions of (extracted) sutures and gut lumen, respectively].

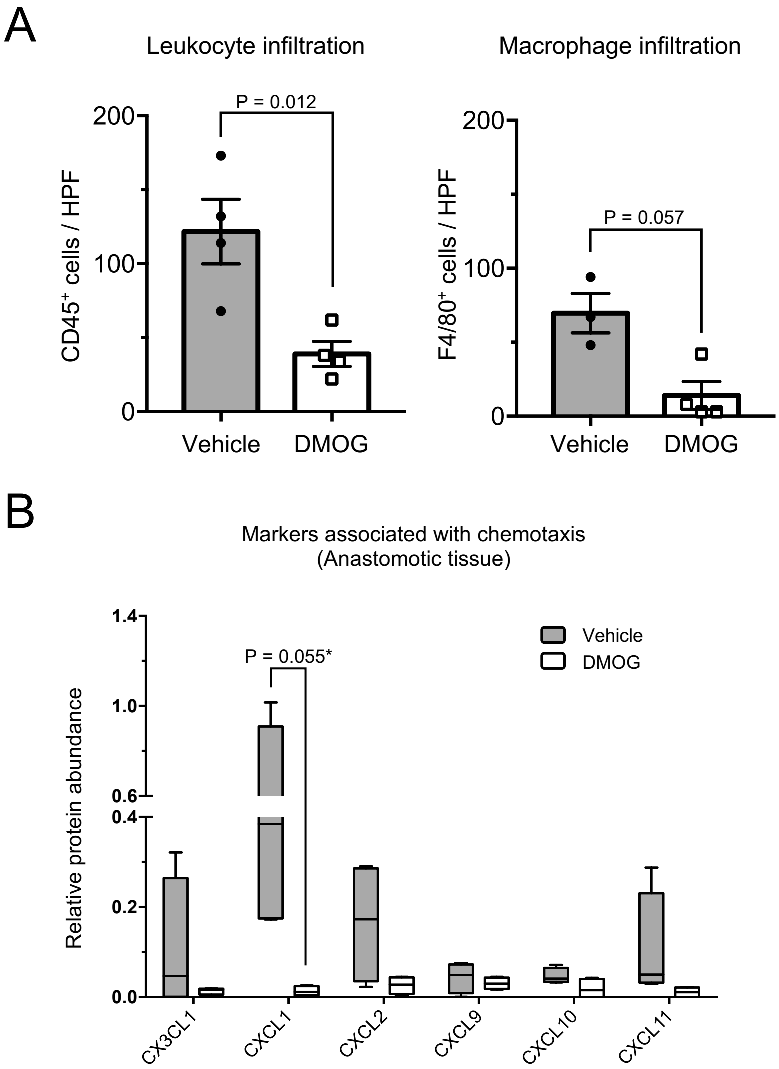

Supp. Figure 7. ***(A)*** Histomorphometric quantification of CD45 (*left*) and F4/80 (*right*) immunostaining revealing decreased leukocyte and macrophage infiltration within DMOG-treated ischemic colonic anastomoses compared to vehicle-treated mice (n = 3-4 animals per group; Mann-Whitney U or Student's t test where appropriate). ***(B)*** Antibody array, analysing the relative protein abundance of chemotaxis-associated cytokines within whole tissue from vehicle- and DMOG-treated ischemic colonic anastomoses (n = 4 biological replicates; *non-adjusted P-value reported, after correction for multiple comparison P = 0.289).

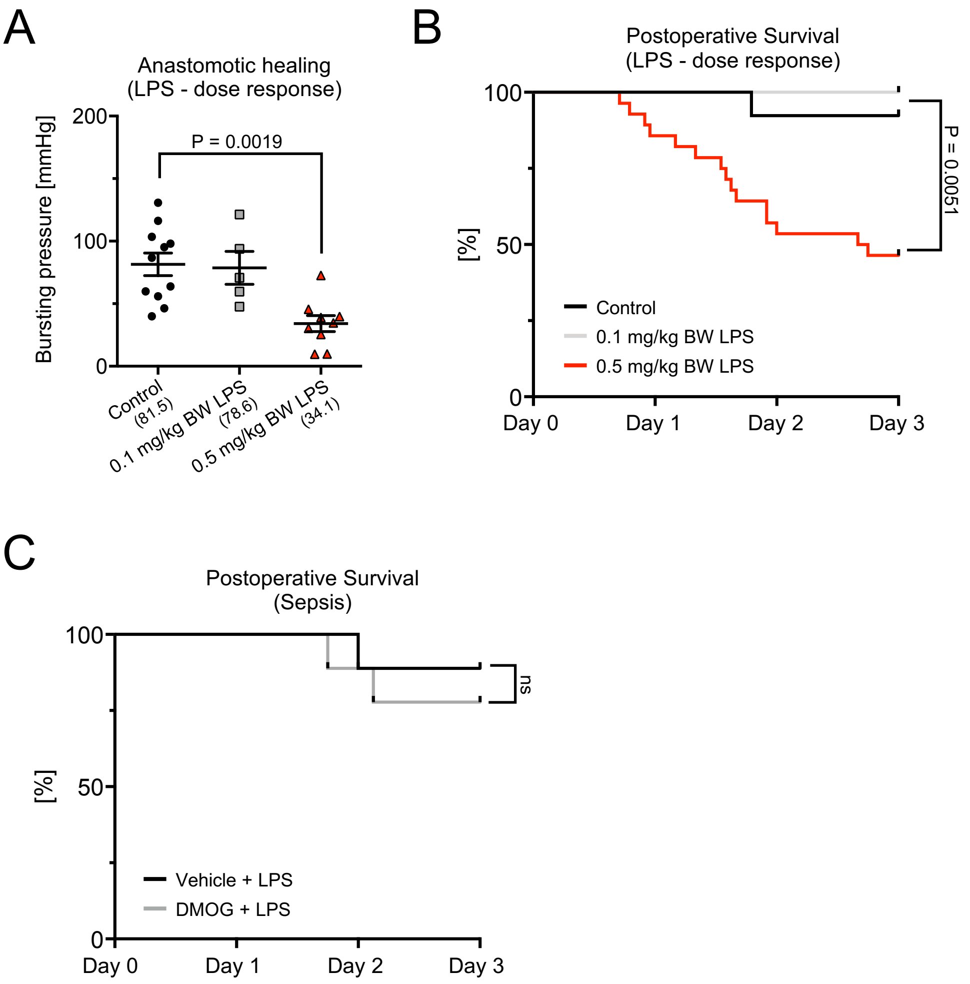

Supp. Figure 8. ***(A + B)*** Bursting pressure analysis (*A*) of healthy and septic colonic anastomoses on day 3 and postoperative survival analysis (*B*) of mice treated with vehicle or different dosages of LPS (0.1 versus 0.5mg/kg BW) 18h before creation of colonic anastomoses [n = 5-11 (in *a*) and 5-28 (in *B*) animals per group; differences in bursting pressure (in *A*) were analysed by ANOVA with post-hoc test, survival curves (in *B*) by log-rank test]. Note, that only high dosages (i.e., 0.5mg/kg BW) of LPS significantly reduced the bursting pressure (*A*) and postoperative survival (*B*), suggesting impaired anastomotic healing in those mice. ***(C)*** Analysis of postoperative survival showing no significant effect of DMOG on survival after LPS-induced septic colonic anastomoses (n = 9 animals per group; survival curves by log-rank test, P = 0.541).

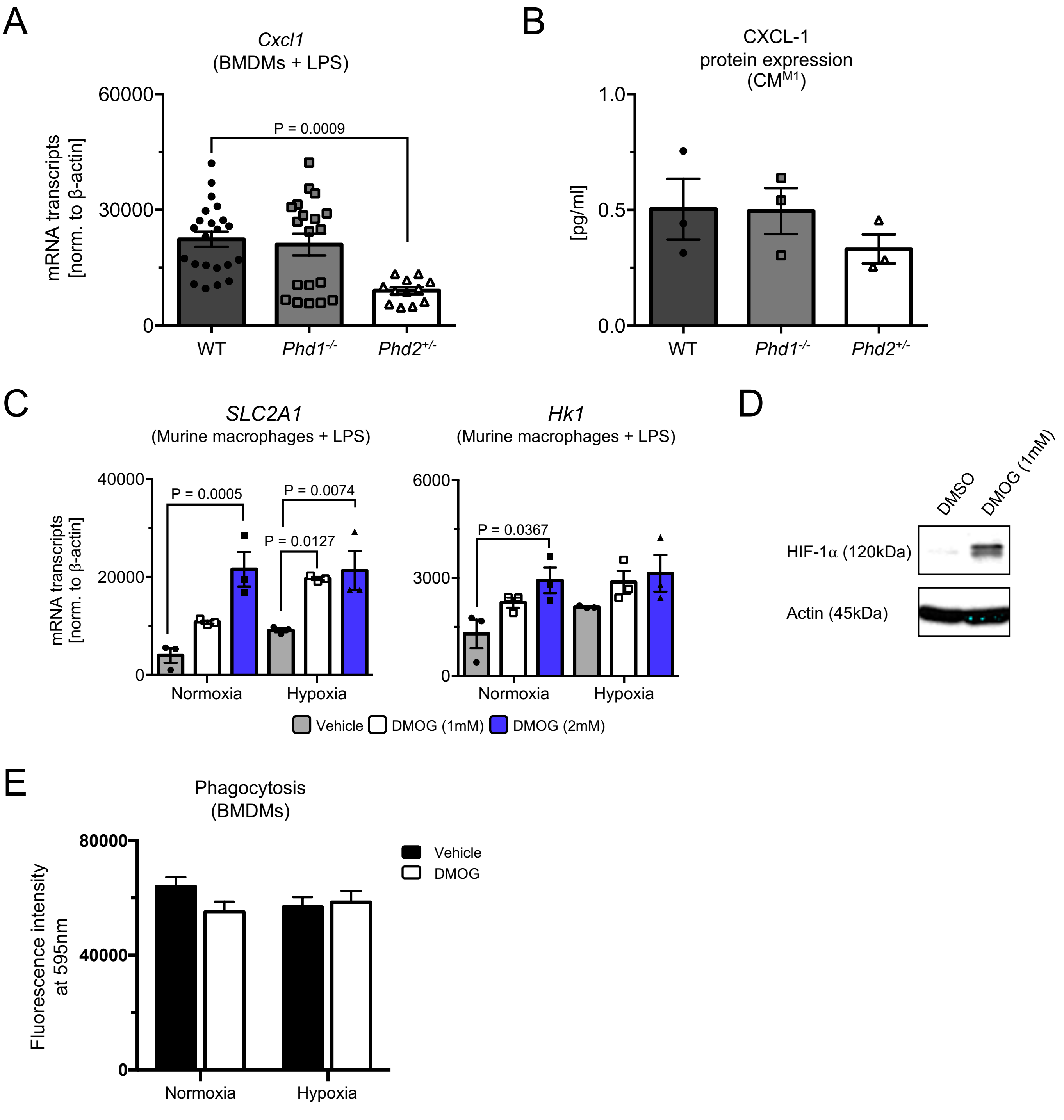

Supp. Figure 9. ***(A + B)*** Real-time PCR analysis (*A*) and ELISA (*B*) showing decreased mRNA and protein levels of chemotactic cytokines C-X-C motif chemokine ligand 1 (*Cxcl1*) in *Phd2*$^{+/-}$ bone marrow-derived macrophages (BMDMs) compared to WT and *Phd1*$^{-/-}$ mice (n = 3, pooled data from individual biological replicates; ANOVA with post-hoc test]. ***(C)*** Real-time PCR analysis of HIF-target genes Solute Carrier Family 2 Member 1 (*Slc2a1*, also known as GLUT-1; *left graph*) and hexokinase 1 (*right graph*) in vehicle- or DMOG-treated (1mM or 2mM) murine macrophages (J774A.1 cell line) treated 24h with normoxia (21% oxygen) or hypoxia (0.75% oxygen) +/- LPS (100ng/ml) to induce macrophage polarization. ANOVA with post-hoc test (GLUT-1) or Kruskal-Wallis with post-hoc test (Hk1). ***(D)*** Analysis of HIF-1α protein expression by western blotting after 3h of vehicle- or DMOG-treatment (1mM) in THP-1 macrophages. Representative images of western blots labelled with actin and HIF-1α. Both analysis of mRNA levels of HIF target genes and HIF-1α protein levels suggest marked up-regulation of the HIF-pathway in DMOG-treated cells. ***(E)*** Quantification of fluorescent bacterial particles (pHrodo) phagocytosed by BMDMs upon treatment with vehicle or DMOG in normoxic and hypoxic culture conditions. All experiments were performed in biological triplicates.

A

Channel	-B525-FITC%	-B610-ECD%	-B690-PC5.5%	-Y585-PE%	-Y610-mCHERRY%	-Y675-PC5%	-Y710-PC5.5%	-Y763-PC7%	-R660-APC%	-R712-APCA700%
B525-FITC		0.00	0.00	0.00	0.00	0.00	0.00	0.00	11.31	0.00
B610-ECD	0.00		0.00	0.00	0.00	0.00	0.00	0.00	0.00	0.00
R660-APC	-0.93	0.00	0.00	0.00	0.00	0.00	0.00	0.00		0.00
R712-AP...	0.00	0.00	0.00	0.00	0.00	0.00	0.00	0.00	0.00	

Channel	-R763-APCA750%	-V450-PB%	-V525-KrO%	-V610%	-V660%	-V763%	-NUV450%	-NUV525%	-NUV675%	-IR840-A790%	-IR885%
B525-FITC	0.00	0.00	0.00	0.00	0.00	0.00	0.00	0.00	0.00	0.00	0.00
B610-ECD	0.00	0.00	0.00	0.00	0.00	0.00	0.00	0.00	0.00	0.00	0.00
R660-APC	0.00	0.00	0.00	0.00	0.00	0.00	0.00	0.00	0.00	0.00	0.00
R712-AP...	0.00	0.00	0.00	0.00	0.00	0.00	0.00	0.00	0.00	0.00	0.00

B

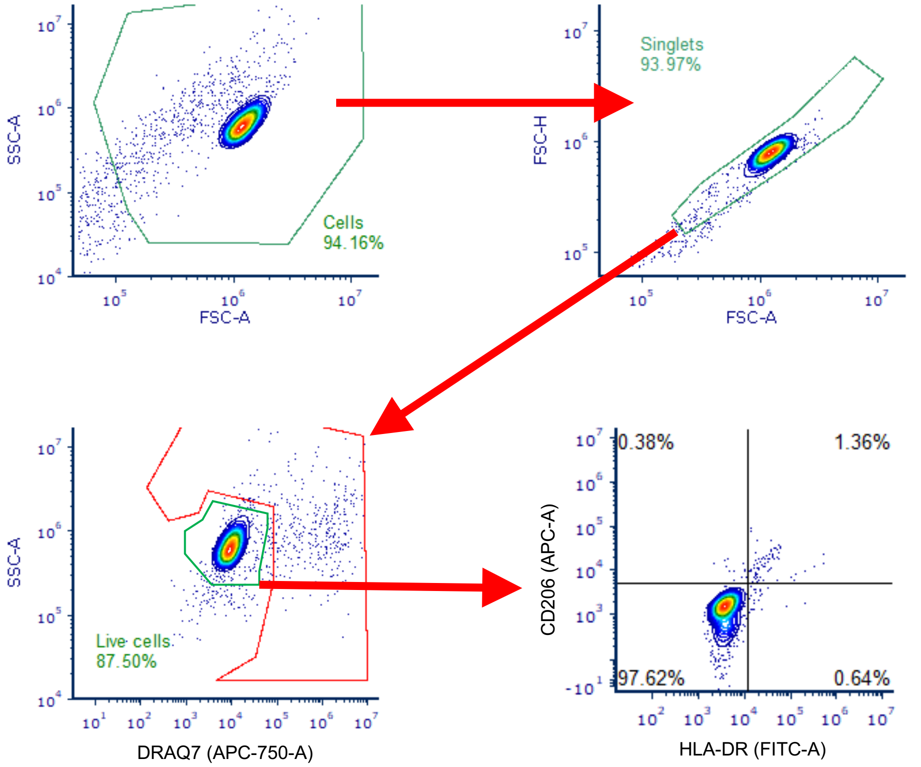

Supp. Figure 10. ***(A)*** Compensation matrix for flow cytometry experiments. ***(B)*** Gating strategy for flow cytometry experiments. Utilizing scatter characteristics [side scatter (SSC) area (A), forward scatter (FSC) area (A) and height (H)], and DRAQ7-labelling viable, single cells were gated and then further analyzed regarding their surface protein expression of the M1 marker HLA-DR (FITC) and the M2 marker CD206 (APC).
